# In Vitro Digestive-Enzyme Inhibition and Antiglycation Activity of *Cannabis sativa* L. Essential Oil: Experimental Evaluation and Molecular Docking Analysis

**DOI:** 10.3390/cimb48090938

**Published:** 2026-09-14

**Authors:** Rafik El-Mernissi, Naoual El Menyiy, Rhizlan Abdnim, Oumayma Sayah, Yahya El-Mernissi, Aziz Zouhri, Mohamed Chebaibi, Moneerah J. Alqahtani, Jawaher H. Alqahtani, Joe Miantezila Basilua, Oualid Abboussi, Lhoussain Hajji

**Affiliations:** 1 Humain Nutrition, Bioactives and Oncogenetics Team, Faculty of Sciences, Moulay Ismail University, Meknes 50003, Moroccol.hajji@umi.ac.ma (L.H.); 2 Physiology and Physiopathology Team, Faculty of Sciences, Genomic of Human Pathologies Research Centre, Mohammed V University, Rabat 1014, Morocco; o.abboussi@um5r.ac.ma; 3 Laboratory of Pharmacology, National Agency for Medicinal and Aromatic Plants, Taounate 34025, Morocco; 4 Laboratory of Bioresources, Biotechnology, Ethnopharmacology and Health, Department of Biology, Faculty of Sciences, Mohammed First University, Oujda 60000, Morocco; 5 Physical Chemistry of Natural Substances and Process Research Team, Laboratory of Applied Chemistry and Environment, Faculty of Sciences, Chemistry Department, Mohammed First University, Oujda 60000, Morocco; 6 Applied Chemistry Research Unit, Faculty of Science and Techniques, Abdelmalek Essaadi University, Al-Hoceima 32000, Morocco; 7 Higher Institute of Nursing Professions and Health Techniques, Fez 30033, Morocco; 8 Biomedical and Translational Research Laboratory, Faculty of Medicine and Pharmacy of Fez, Sidi Mohamed Ben Abdellah University, Fez 30000, Morocco; 9 Department of Pharmacognosy, College of Pharmacy, King Saud University, P.O. Box 2457, Riyadh 11451, Saudi Arabia; 10 Department of Biostatistics and Mathematics, Faculty of Pharmacy, Paris Cité University, 4, Avenue de Observatories, 75006 Paris, France; joe.miantezila-basilua@u-paris.fr

**Keywords:** *Cannabis sativa* L., essential oil, digestive-enzyme inhibition, α-amylase, α-glucosidase, pancreatic lipase, antiglycation activity, SDG3, molecular docking, ADME analysis

## Abstract

**Aim of the Study**: The aim of this study was to characterize the volatile phytochemical composition of *Cannabis sativa* L. essential oil (CSEO) and evaluate its in vitro inhibitory activity against selected digestive enzymes and its antiglycation activity. Molecular docking and ADMET analyses were additionally performed to explore the possible interactions and predicted pharmacokinetic properties of the major identified constituents. **Materials and Methods**: The chemical composition of CSEO was characterized by Gas Chromatography–Mass Spectrometry (GC-MS). Its in vitro inhibitory activity was evaluated against α-amylase, α-glucosidase, and pancreatic lipase, while its antiglycation activity was assessed by monitoring the formation of glycation products at different stages of the glycation process. Molecular docking simulations were performed to investigate the potential interactions of major identified terpenes with the active sites of the investigated enzymes. In addition, ADME analysis was conducted to predict selected pharmacokinetic and drug-likeness properties of the major constituents. **Results**: gc-ms analysis revealed a complex terpenoid profile, with β-caryophyllene as the major constituent, followed by selina-3,7(11)-diene (8.00%), cubenol (6.62%), γ-eudesmol (6.57%), epiglobulol (6.04%), and valencene (5.80%). CSEO showed significant in vitro inhibitory activity against α-amylase, α-glucosidase, and pancreatic lipase. The essential oil also exhibited antiglycation activity, with inhibition observed at different stages of glycation-product formation. Molecular docking analysis indicated that several major sesquiterpenes could interact with the investigated enzyme targets through different binding interactions. ADME analysis provided predicted pharmacokinetic and drug-likeness profiles for the major constituents. **Conclusions**: The findings demonstrate that *Cannabis sativa* L. essential oil possesses in vitro digestive-enzyme inhibitory and antiglycation activities. The docking results provide molecular-level insights into possible interactions between major essential-oil constituents and the investigated enzyme targets. These findings support further investigation of CSEO and its major constituents using appropriate in vivo and mechanistic studies to determine their biological relevance and potential applications.

## 1. Introduction

The global epidemic of diabetes mellitus, particularly Type 2 Diabetes Mellitus (T2DM), represents one of the most pressing metabolic threats of the 21st century. According to the International Diabetes Federation (IDF), the global diabetic population reached 425 million in 2017 and is projected to surge to 629 million by 2045 [1]. A central clinical hallmark of T2DM is acute postprandial hyperglycemia rapid, transient spikes in blood glucose following meals which accelerates systemic vascular damage, promotes chronic low-grade inflammation, and exhausts pancreatic beta-cell function [2]. Managing postprandial glucose and lipid absorption by inhibiting key digestive hydrolases, specifically alpha-amylase, alpha-glucosidase, and pancreatic lipase, constitutes a primary therapeutic avenue for regulating dietary glucose influx and suppressing hyper-lipidemia induced visceral obesity [3,4]. Simultaneously, sustained exposure to elevated glucose promotes non-enzymatic protein glycation, generating reactive oxygen species (ROS) and accelerating the formation of irreversible advanced glycation end-products (AGEs) [5,6]. The tissue accumulation of AGEs induces structural protein dysfunction, cross-linking, and downstream inflammatory signalling via the AGE-RAGE axis, driving severe diabetic micro- and macro-vascular sequelae, including retinopathy, neuropathy, and nephropathy [7,8].

Although pharmacological interventions such as insulin and synthetic oral hypoglycemic agents (acarbose, miglitol, and voglibose) effectively suppress key digestive enzymes, their long-term clinical utility is frequently constrained by undesirable adverse effect profiles, encompassing gastrointestinal distress, abdominal cramping, flatulence, hepatorenal physiological disturbances, and weight gain [9,10]. Consequently, there is an urgent demand for safer, multi-targeted natural alternatives capable of regulating postprandial glycemic spikes while concurrently shielding systemic macromolecules from oxidative glycation damage.

Unlike synthetic monospecific pharmaceuticals, plant-derived natural matrices exert their therapeutic efficacy against metabolic syndrome through complex polypharmacological and complementary mechanisms [11,12]. Phytoconstituents operate within a multi-target therapeutic network, at the luminal level, specialized secondary metabolites competitively or non-competitively modulate alpha-amylase, alpha-glucosidase, and pancreatic lipase to flatten postprandial glucose and lipid absorption curves. Simultaneously, specific plant bioactives enhance the endogenous incretin effect by stimulating intestinal L-cell glucagon-like peptide-1 (GLP-1) secretion and inhibiting dipeptidyl peptidase-4 (DPP-4) degradation [9,12,13]. Systemically, co-existing antioxidants stimulate glucose-stimulated insulin secretion (GSIS) and foster pancreatic beta-cell proliferation and survival, while trapping reactive dicarbonyl intermediates (methylglyoxal) to halt protein carbonylation and AGE assembly [14,15]. Harmonizing luminal digestive enzyme retardation, incretin stabilization, beta-cell protection, and systemic antiglycation provides a synergistic paradigm that mitigates metabolic resistance and minimizes individual compound toxicity [16,17].

Within the Mediterranean basin, Morocco represents a major biodiversity hotspot characterized by exceptional floristic wealth and a deeply rooted ethnopharmacological heritage [18,19] the traditional Moroccan pharmacopeia relies heavily on endemic aromatic and medicinal plants to manage metabolic disorders, providing a continuous source of novel bioactive leads [20,21]. Among the prominent species of the Moroccan flora, *Cannabis sativa* L. recently integrated into a formal legal regulatory framework in Morocco for medical and industrial applications occupies a unique ethnopharmacological position. Beyond its well-documented cannabinoid profile *C. sativa* synthesizes over 480 chemical constituents, including more than 140 distinct volatile mono- and sesquiterpenes that constitute the predominant chemical matrix of its essential oil [22]. Terpenes represent a structurally diverse class of secondary plant metabolites ubiquitously present in aromatic essential oils, with emerging experimental literature highlighting their significant hypoglycemic, antioxidant, and antiglycation capacities [23,24]. While ethnopharmacological surveys document the traditional use of *Cannabis* preparations in managing metabolic and inflammatory symptoms [25,26]. The pharmacological capacity of its non-cannabinoid, terpene-rich essential oil to simultaneously attenuate digestive hydrolases and protect against multi-stage protein glycation remains largely uncharacterized. The rationale for selecting CSEO as a candidate for these biological targets is based on the chemical nature of its main terpene constituents. Monoterpenes and sesquiterpenes commonly found in *Cannabis* essential oils, such as β-caryophyllene, myrcene, α-pinene, and limonene, are small, lipophilic molecules that can easily interact with hydrophobic regions of enzyme active sites. Previous studies have shown that these terpenes may bind to α-amylase and α-glucosidase through hydrophobic interactions, van der Waals forces, and, in some cases, hydrogen bonding with nearby amino acid residues [27,28,29]. Such interactions can affect enzyme activity and substrate binding. In addition, several terpenes have been reported to show antioxidant and carbonyl-scavenging properties, which may help reduce oxidative stress and slow down non-enzymatic protein glycation [30,31]. In particular, their ability to interact with reactive dicarbonyl compounds like methylglyoxal has been suggested as a possible way to slow the Maillard reaction and the formation of AGEs [30,32]. Overall, these combined enzyme-inhibiting and antioxidant properties provide a clear scientific basis for studying the terpene-rich CSEO against digestive enzymes and protein glycation. However, these findings are based on previous reports and do not confirm that specific compounds are responsible for the observed activity, which must be verified experimentally.

To date, most pharmacological research on *Cannabis sativa* L. has focused almost exclusively on non-volatile cannabinoids and their interactions with central cannabinoid receptors. Consequently, the therapeutic potential of the volatile, terpene-rich essential oil fraction particularly from newly regulated Moroccan crops in modulating metabolic digestive enzymes and protecting against macromolecular protein oxidation remains largely unaddressed.

Therefore, the primary objective of this work was to identify and quantify the volatile terpene constituents of CSEO using gas chromatography-mass spectrometry (GC-MS) and evaluate its concentration-dependent inhibitory kinetics against key enzymes regulating postprandial carbohydrate and lipid absorption (alpha-amylase, alpha-glucosidase, and pancreatic lipase). Furthermore, this study aimed to investigate the protective efficacy of CSEO across early, intermediate, and late stages of non-enzymatic protein glycation by systematically assessing fructosamine content, protein carbonyl group formation, amyloid beta-structure aggregation via the Congo Red assay, and total fluorescent AGE accumulation.

## 2. Materials and Methods

### 2.1. Plant Material and Essential Oil Extraction

*Cannabis sativa* L. plant material consisted of female plants of the Beldiya chemotype, collected in September 2021 at the flowering stage from the Tafrant region, Taounate Province, Morocco (34°39′28.4″ N, 5°05′58.9″ W). Collection, transport, and subsequent biological assessments were conducted under official authorization (Ref. 10/ANRAC). Botanical identification was performed by Dr. Mohammed Taleb, botanist at the Scientific Institute of Rabat, Morocco, and a voucher specimen was deposited in the institute’s herbarium under reference number RAB 112735. Fresh leaves and inflorescences were subjected to hydro-distillation for 3 h using a Clevenger apparatus, with 200 g of fresh plant material distilled with 1.5 L of water for each extraction. The essential oil yield was 0.09 ± 0.02% (*v*/*w*). The obtained essential oil was dehydrated over anhydrous sodium sulfate (Na_2_SO_4_), filtered, transferred into sealed vials, and stored at 4 °C until further analysis.

### 2.2. Chemical Characterization

The analysis of the extracted essential oils (CSEO) was conducted using gas chromatography (Shimadzu GC-2010, Kyoto, Japan). This instrument features a capillary column RTX-5 (5% diphenyl, 95% dimethylpolysiloxane, dimensions: 30 × 0.25 mm, film thickness: 0.25 µm) and is coupled with a mass spectrometer detector (QP2010-MS) (Shimadzu Corporation, Kyoto, Japan). Helium gas served as the carrier gas, maintained at a constant pressure of 100 KPa. Initially, the oven temperature was set to 50 °C for approximately 1 min, followed by a gradient increase of +10 °C until reaching 250 °C, which was then maintained for 1 min. For qualitative and semi-quantitative analysis, a solution of 1 µL of the sample prepared in hexane (at a concentration of 50 mg g^−1^) was injected in split mode (split ratio = 50–80), and the GC-MS system operated in scan mode. Identification of the chemical composition of the samples relied on comparing their mass spectra with data stored in the National Institute of Standards and Technology (NIST147) database. The data collection was performed using LabSolutions version 2.5. An *n*-alkane series (C8-C20) processed under uniform experimental conditions served as an external standard for retention index calculations. Identification was confirmed by comparing these experimental RIs with published database values (NIST Chemistry WebBook and Adams Library) specific to low-polarity stationary phases (Rtx-5, HP-5, or DB-5).

### 2.3. In Vitro α-Amylase Inhibitory Assay

α-Amylase inhibitory activity was evaluated according to Abdnim et al. (2023) with slight modifications [33]. Reaction mixtures contained 100 µL phosphate buffer (200 mM, pH 6.9), 100 µL α-amylase solution (13 IU), and 100 µL of either *Cannabis sativa* Lessential oil (CSEO; 0.1–1 mg/mL) or acarbose (0.1–1 mg/mL). For the enzymatic inhibition assays, CSEO was tested over a concentration range of 0.06, 0.125, 0.25, 0.5, and 1 mg/mL After 10 min preincubation at 37 °C, enzymatic hydrolysis was initiated by adding 100 µL starch solution (1% *w*/*v* in phosphate buffer) followed by 20 min incubation at 37 °C. Reactions were terminated with 300 µL 3,5-dinitrosalicylic acid (DNS) reagent. Samples were vortexed, heated at 100 °C for 8 min for chromogenic development, then immediately cooled in an ice-water bath (5 min). Following dilution with 1 mL distilled water, absorbance was quantified at 540 nm. Inhibition percentages were calculated as:

$$\%\;Inhibition=\left[\frac{\left(Abs\;Control-Abs\;Samples\right)}{\left(Abs\;Control\right)}\right]\times 100$$

IC_50_ values were derived from dose-inhibition curves generated using nonlinear regression analysis in GraphPad Prism (v.9.0).

### 2.4. In Vitro α-Glucosidase Inhibitory Assay

α-Glucosidase inhibitory activity was assessed according to Elrherabi et al. method [34]. using reaction mixtures containing 1.0 mL phosphate buffer (pH 7.5), 0.1 mL α-glucosidase solution (10 IU), and 200 μL of either test samples (CSEO: 0.1–1 mg/mL), positive control (acarbose: 0.1–1 mg/mL), or negative control (distilled water). For the enzymatic inhibition assays, CSEO was tested over a concentration range of 0.06, 0.125, 0.25, 0.5, and 1 mg/mL Following 20 min preincubation at 37 °C, enzymatic hydrolysis was initiated with 0.1 mL sucrose solution and terminated after 5 min at 100 °C. Subsequently, 1.0 mL GOD-POD reagent (glucose oxidase-peroxidase) was added, followed by 10 min incubation at 37 °C.

CSEO was dispersed in the aqueous buffer using Tween 80 as an emulsifying agent and vortexed thoroughly before dilution to the desired concentrations Absorbance was measured at 500 nm, with percentage inhibition calculated as:

$$\%\;Inhibition=\left[\frac{\left(Abs\;Control-Abs\;Samples\right)}{\left(Abs\;Control\right)}\right]\times 100$$

The half-maximal inhibitory concentration (IC_50_) was calculated through curve fitting of concentration-response data, performed with GraphPad Prism software (Version 9.0).

### 2.5. In Vitro Lipase Inhibitory Assay

Pancreatic lipase inhibitory activity was assessed according to haddou et al. [35] using p-nitrophenyl palmitate (pNPP) as substrate. Stock solutions comprised CSEO (1000 μg/mL in Tris-HCl buffer, pH 8.0), porcine pancreatic lipase (1 mg/mL in Tris-HCl, pH 8.0), and pNPP substrate (30.2 mM in acetonitrile) Test concentrations (0.125–1 mg/mL) were prepared through serial dilution of the CSEO stock. Test concentrations (0.125–1 mg/mL) were prepared through serial dilution of the CSEO stock. Assay mixtures containing 700 μL Tris-HCl buffer (pH 8.0), 100 μL lipase solution, and 200 μL diluted extract were preincubated at 37 °C for 15 min. Enzymatic reactions were initiated by adding 100 μL pNPP solution followed by 30 min incubation at 37 °C. Absorbance was measured spectrophotometrically at 410 nm, with orlistat serving as positive control at identical concentrations. CSEO and orlistat were dispersed in the aqueous buffer using Tween 80 as an emulsifying agent and vortexed thoroughly before dilution to the desired concentrations. All analyses were performed in triplicate, and percentage inhibition was calculated as:

$$\%\;Inhibition=\left[\frac{\left(Abs\;Control-Abs\;Samples\right)}{\left(Abs\;Control\right)}\right]\times 100$$

The concentration of oil required for 50% inhibition of lipase activity (IC_50_) was interpolated from a dose-response curve plotting percent inhibition against oil concentration (mg/mL).

### 2.6. Determination of Antiglycation Activity

Evaluation of antiglycation activity followed an established hemoglobin-glucose assay [36], with modifications. Briefly, glycation was induced by incubating hemoglobin (1%, 1 mL), glucose (4 mg/mL, 1 mL), and gentamicin (40 mg/mL, 5 µL) in phosphate buffer (pH 7.4) with test samples (CSEO, 1 mL) at 37 °C for 72 h. Gallic acid was used as the reference inhibitor in the positive control. Absorbance of the glycation products was quantified at 443 nm (triplicate measurements). The percentage inhibition relative to the appropriate control was calculated as follows:

$$\%\;Inhibition=\left[1-\left(DO\;Blanc-\frac{\left(DO\;Control-DO\;Test\right)}{\left(DO\;Control\right)}\right)\right]\times 100$$

### 2.7. In Vitro Glycation of Albumin Assay

Antiglycation activity against albumin glycation was assessed using a modified method based on [37]. Bovine serum albumin (BSA, 10 mg/mL) was dissolved in 100 mM potassium phosphate buffer (pH 7.4) containing 0.2% sodium azide. Reaction mixtures contained 1 mL BSA solution, 500 mM glucose, and either the CSEO or aminoguanidine (positive control, dissolved in PBS). Samples were incubated at 37 °C in the dark for 1 week. Advanced glycation end-product (AGE) formation was quantified by fluorescence spectroscopy (excitation 355 nm, emission 460 nm; Horiba FluoroMax-4, Kyoto, Japan). Percent inhibition relative to the control was calculated for each test sample.

#### 2.7.1. Determination of Fructosamine Levels

A thiobarbituric acid (TBA) assay, based on Elrherabi [38] protocol, was employed to quantify fructosamine levels. Briefly, glycated BSA was reacted with 0.05 M TBA under heat (95–100 °C, 20 min). Absorbance of the cooled reaction mixtures was read at 443 nm. Percent inhibition relative to the positive control was determined as:

$$\%\;Inhibition=\left[\frac{\left(A0-A1\right)}{\left(A0\right)}\right]\times 100$$

where A0 represents the positive control absorbance (glycated BSA + TBA) and A1 represents the test sample absorbance (glycated BSA + TBA + CSEO).

#### 2.7.2. DNPH Colorimetric Assay for Protein Carbonyl Determination

Albumin carbonyl content was quantified using a modified 2,4-dinitrophenylhydrazine (DNPH) assay based on Abdnim et al. [37] method Glycated samples (0.5 mL) were reacted with an equal volume of 10 mM DNPH in 2.5 M HCl for 1 h at ambient temperature. Absorbance was measured at 365 nm, and carbonyl concentration was calculated using the molar absorptivity coefficient of DNPH (365 nm = 21 mM^−1^cm^−1^). Percentage inhibition of carbonyl formation was determined as:

$$\%\;inhibition=\left[1-\left[\;\frac{\left(A0\;-\;A1\right)}{(A0)\;}\;\right]\right]\times \;100$$

where:

A_0_ = Absorbance of positive control (glycated albumin + DNPH).

A_1_ = Absorbance of test sample (glycated albumin + DNPH + CSEO).

#### 2.7.3. Quantification of β-Amyloid Aggregation

Quantification of β-amyloid aggregation inhibition was performed using a Congo red (CR) spectral shift assay adapted from El rherabi et al. [38]. Reaction mixtures containing glycated albumin (0.5 mL) and CR solution (100 μM in PBS-ethanol [9:1 *v*/*v*], pH 7.4; 100 μL) were incubated for 20 min at 25 °C. Absorbance measurements at 530 nm were acquired using a spectrophotometer. The percentage inhibition of amyloid fibrillization was calculated as:

$$\%\;of\;inhibition=\left[1-\left[\;\frac{\left(A0\;-\;A1\right)}{(A0)\;}\;\right]\right]\times \;100$$

#### 2.7.4. Measurement of Total Fluorescent AGEs

Quantification of total advanced glycation end products (AGEs) was performed by monitoring fluorescence intensity of glycated albumin samples adapted from [37]. Reaction mixtures containing glycated albumin with or without inhibitor (CSEO) were analyzed using a fluorescence spectrophotometer with excitation at 370 nm and emission at 440 nm. Percentage inhibition of AGE formation was calculated as:

$$\%\;of\;inhibition=\left[\frac{\left(F0\;-\;F1\right)}{(F0)\;}\right]\times \;100$$

F0 = Fluorescence intensity of positive control (glycated albumin).

F1 = Fluorescence intensity of test sample (glycated albumin + CSEO).

### 2.8. Molecular Docking

#### 2.8.1. Computational Docking Analysis and Rationale for Target Selection

This investigation utilized computational docking to assess the binding interactions of a series of natural compounds from *Cannabis sativa* L. Essential Oil with two protein targets implicated in the management of type 2 diabetes: Alpha-Amylase and Alpha-Glucosidase. The simulations were performed using Maestro 11.5 software (Schrödinger suite) to predict the affinity of these compounds for the enzymes, in order to evaluate their potential inhibitory and antidiabetic activity.

The two protein targets were selected because they represent complementary catalytic steps in the enzymatic hydrolysis of dietary carbohydrates. α-Amylase catalyzes the initial endohydrolysis of internal α-1,4-glycosidic bonds in starch and related polysaccharides, whereas α-glucosidase catalyzes the subsequent hydrolysis of terminal α-glucosidic linkages to release glucose. Evaluating both enzymes therefore provides a mechanistically coherent model for examining whether the identified constituents may interact with distinct stages of carbohydrate digestion rather than relying on a single protein target.

The selected structures, 1B2Y for human pancreatic α-amylase and 5NN8 for human lysosomal acid α-glucosidase, were chosen because both are experimentally determined X-ray structures containing co-crystallized acarbose. The acarbose-bound conformations provided a common structural reference for defining the carbohydrate-binding pockets and comparing the predicted ligand interactions. However, these interactions remain computational predictions and do not constitute direct evidence of biological binding or enzyme inhibition.

#### 2.8.2. Protein Structure Preparation

The three-dimensional crystallographic structures for the target enzymes, Alpha-amylase (PDB: 1B2Y) and alpha-glucosidase (PDB: 5NN8), were retrieved from the RCSB Protein Data Bank. Protein preparation was performed using the Protein Preparation Wizard of Schrödinger-Maestro v11.5. The procedure involved assigning appropriate bond orders, adding hydrogen atoms to heavy atoms, converting selenomethionine residues to methionine, optimizing the orientation of hydroxyl groups, and removing non-essential water molecules and co-crystallized ligands. Water molecules forming fewer than three hydrogen bonds with non-water atoms were also removed. Subsequently, the hydrogen-bonding network was optimized, and a restrained energy minimization was performed using the OPLS3 force field, with the maximum heavy-atom RMSD (root mean square deviation) restrained to 0.30 Å, to relieve steric clashes while preserving the overall protein structure [39].

#### 2.8.3. Ligand Preparation

The ligands corresponding to the compounds identified in Cannabis sativa L. essential oil were downloaded in SDF format from the PubChem database and subsequently processed using the LigPrep module of the Schrödinger Suite. The two-dimensional structures were converted into three-dimensional models, with possible tautomers and ionization states generated at a physiological pH of 7.0 ± 2.0. Correct stereochemical configurations were assigned, and geometry optimization was performed using the OPLS3 force field to generate low-energy three-dimensional conformations suitable for molecular docking. Furthermore, LigPrep was configured to generate a maximum of 32 stereoisomeric forms for each ligand to account for possible stereochemical variations [40].

#### 2.8.4. Receptor Grid Generation

For each enzyme, a grid specifying the potential binding site was created using the Glide module. The grid box had a volumetric spacing of 20 × 20 × 20, and the coordinates were x: 18.935, y: 5.717, z: 46.903 for alpha-amylase, and x: −14.02, y: −38.177, z: 95.206 for alpha-glucosidase [41].

#### 2.8.5. Docking Protocol

Ligand docking was executed using Glide in Standard Precision (SP) mode. The generated poses were ranked according to the Glide gscore (kcal/mol), which provides an estimate of the binding affinity. Analysis of the top-ranked poses focused on pinpointing key molecular interactions, such as hydrogen bonds, hydrophobic contacts, and pi-pi stacking, that stabilize the ligand within the binding site [42].

#### 2.8.6. Docking Protocol Validation

The docking protocol was validated by redocking the co-crystallized ligand, Acarbose, into the active site of each enzyme. Acarbose was extracted from the crystallographic structure and subsequently redocked into the same binding pocket. The reliability of the docking protocol was assessed by calculating the Root Mean Square Deviation (RMSD) between the redocked pose and the original crystallographic conformation of Acarbose. The docking protocol was considered successfully validated when the RMSD value was below 2.0 Å, indicating that the docking procedure could accurately reproduce the experimentally observed binding mode of the co-crystallized ligand.

### 2.9. ADME Prediction

Ligand refinement was carried out by assessing their pharmacokinetic profiles, including absorption, distribution, metabolism, and excretion (ADME) parameters. This step also ensured compliance with Lipinski’s Rule of Five, utilizing the QikProp module integrated into Maestro (version 11.5, Schrödinger Suite). The screening focused on the physicochemical and pharmacokinetic features of the compounds investigated. Key criteria included molecular weight (mol MW), counts of hydrogen bond donors (donorHB) and acceptors (accptHB), total solvent-accessible surface area (SASA), octanol/water partition coefficient (QPlogPo/w), aqueous solubility (QPlogS), Caco-2 cell permeability (QPPCaco), brain/blood partition coefficient (QPlogBB), skin permeability coefficient (QPlogKp), and the percentage of human oral absorption (Percent Human Oral Absorption) [42].

### 2.10. Statistical Analysis

Experimental results are expressed as mean ± SEM of three technical replicate (*n* = 3). Statistical significance was assessed through *t*-test and two-way analysis of variance (ANOVA), followed by Tukey’s multiple-comparison test with differences between group means considered significant at *p* < 0.01 and *p* < 0.05, Different letters indicate statistically significant differences between groups according to Tukey’s post hoc test. All statistical analyses were performed using GraphPad Prism version 9.5.

## 3. Results and Discussion

### 3.1. Volatile Profile and Major Phytochemical Constituents of CSEO

**Figure 1:**
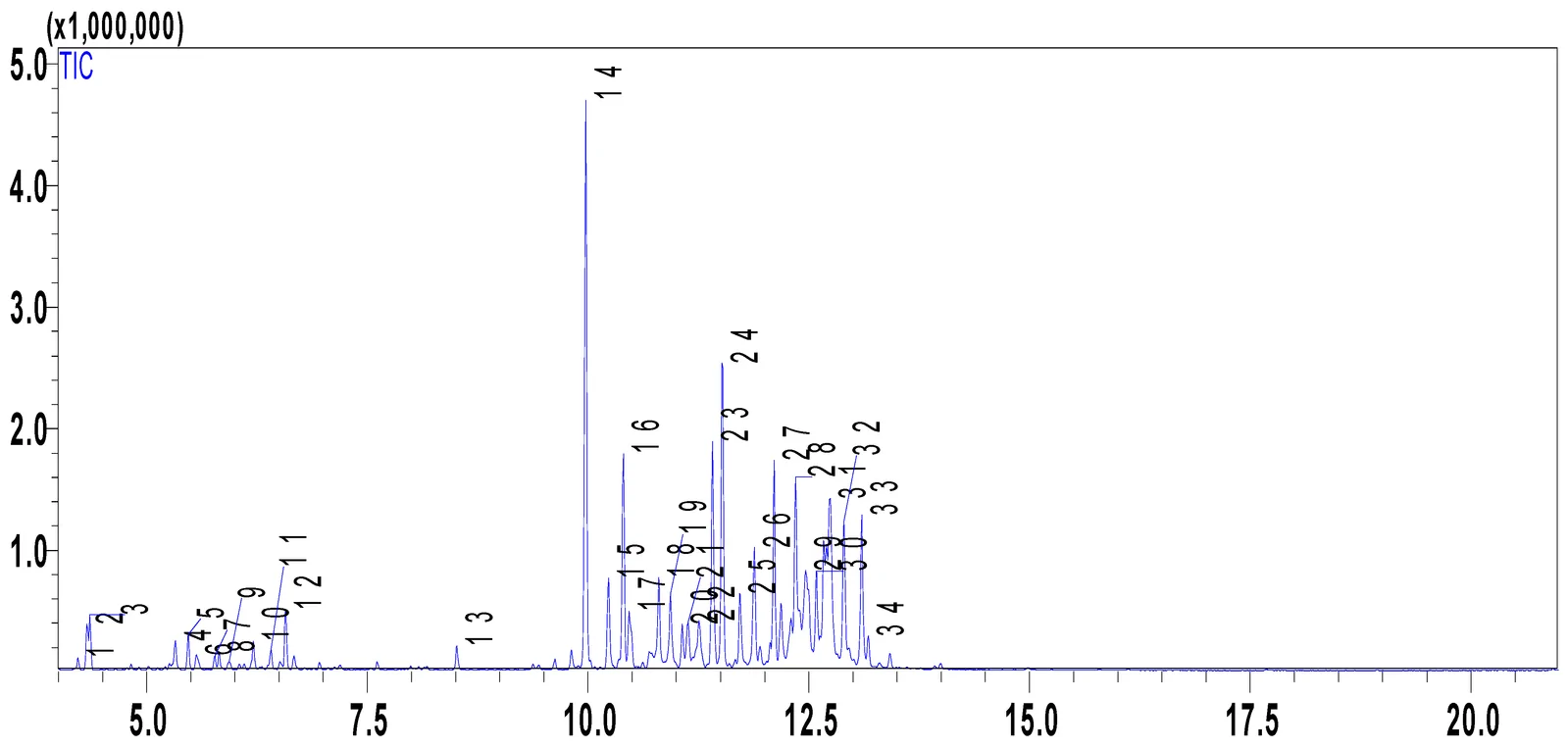
GC-MS chromatogram of CSEO.

The GC–MS analysis of *Cannabis sativa* L. essential oil (CSEO) demonstrated a chemical profile strongly dominated by sesquiterpene-type constituents (34 volatile constituents) (Figure 1, Table 1). Sesquiterpene hydrocarbons (49.78%) represented the major fraction, followed by oxygenated sesquiterpenes (41.14%), while oxygenated monoterpenes (7.81%) and monoterpene hydrocarbons (1.27%) contributed only minor proportions. This compositional pattern is consistent with previous studies reporting the predominance of higher terpenoids in Cannabis essential oils obtained by hydro distillation, where monoterpenes may partially evaporate or degrade due to their higher volatility and thermal sensitivity [43].

Within the sesquiterpene hydrocarbons, β-caryophyllene (13.19%) emerged as the main compound, confirming its widely recognized role as a characteristic and abundant sesquiterpene in *Cannabis sativa* L. [44]. Other significant hydrocarbons included selina-3,7(11)-diene (8.00%), valencene (5.80%), cis-α-bisabolene (5.21%), γ-gurjunene (2.53%), and β-eudesmene (2.87%), all contributing to the typical woody–spicy olfactory profile associated with hemp essential oil. The relative abundance of these compounds indicates a robust activity of sesquiterpene synthases in the plant, reflecting the chemotypic traits of the analyzed material, likely influenced by genetics, developmental stage, and environmental conditions [45]. In addition, the composition of the essential oil is strongly affected by the extraction process itself [46]; hydro-distillation, in particular, is known to enhance the recovery of sesquiterpenes while promoting partial loss or transformation of more volatile monoterpenes, thereby shaping the final chemical profile [47].

Oxygenated sesquiterpenes formed a substantial portion of the oil. Cubenol (6.62%), γ-eudesmol (6.57%), epiglobulol (6.04%), guaiol (5.58%), and bulnesol (5.27%) were among the major constituents. These compounds are known for their antimicrobial, anti-inflammatory, and antioxidant activities, which increase the potential therapeutic value of the essential oil [48]. The detection of other bioactive oxygenated terpenoids, such as α-bisabolol (4.12%), isoaromadendrene epoxide (3.55%), and α-copaen-11-ol (3.39%), further supports the functional significance of this chemical group.

In contrast, monoterpenes occurred in relatively low proportions. α-Terpineol (1.28%), cineole (1.02%), and D-limonene (0.98%) were the primary representatives. The low monoterpene content may reflect partial volatilization during hydro-distillation or intrinsic cultivar-specific characteristics associated with sesquiterpene-rich chemotypes [49]. Minor monoterpene hydrocarbons such as β-cymene, β-linalool, and α-pinene epoxide were detected below 1%, reinforcing the sesquiterpene-dominant nature of the sample.

Broadly, the essential oil profile characterized in the present work matches trends previously documented for *Cannabis sativa* L. chemotypes enriched in caryophyllene, selinenes, eudesmols, and other sesquiterpenoids. The high proportion of oxygenated sesquiterpenes suggests enhanced pharmacological potential, particularly regarding anti-inflammatory and antimicrobial properties. Differences in abundance compared with other published profiles may be attributed to cultivar genetics, geographic origin, agronomic factors, and post-harvest processing [50,51,52].

**Table 1:** Chemical composition of the CSEO.

Peak	Compound	RT (min)	Area %	Chemical Structure	Chemical Classes	RI (exp)	RI (lit)	ΔRI
1	*β*-Cymene	4.223	0.29	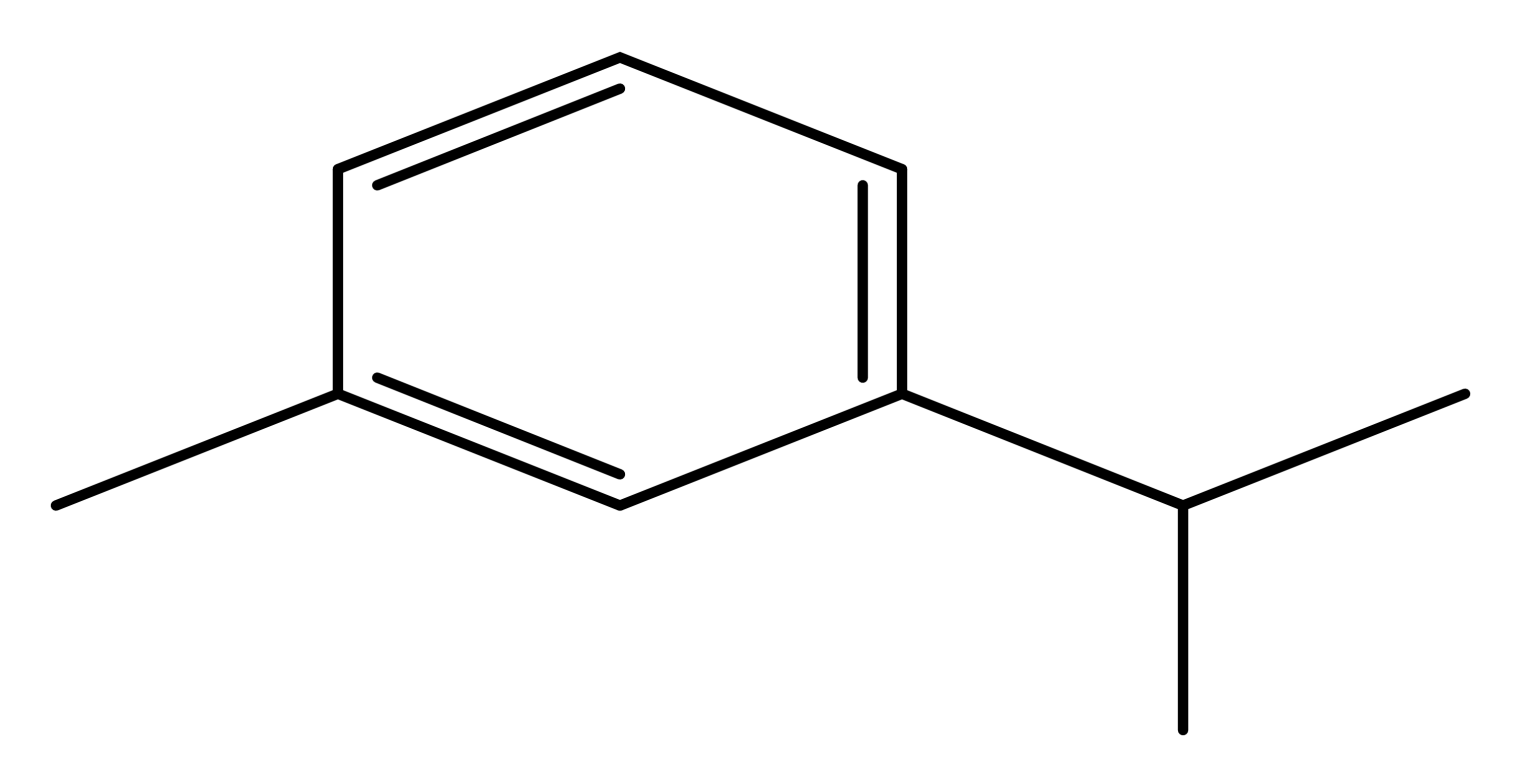	Monoterpene hydrocarbon	1024	1026	2
2	Cineole	4.327	1.02	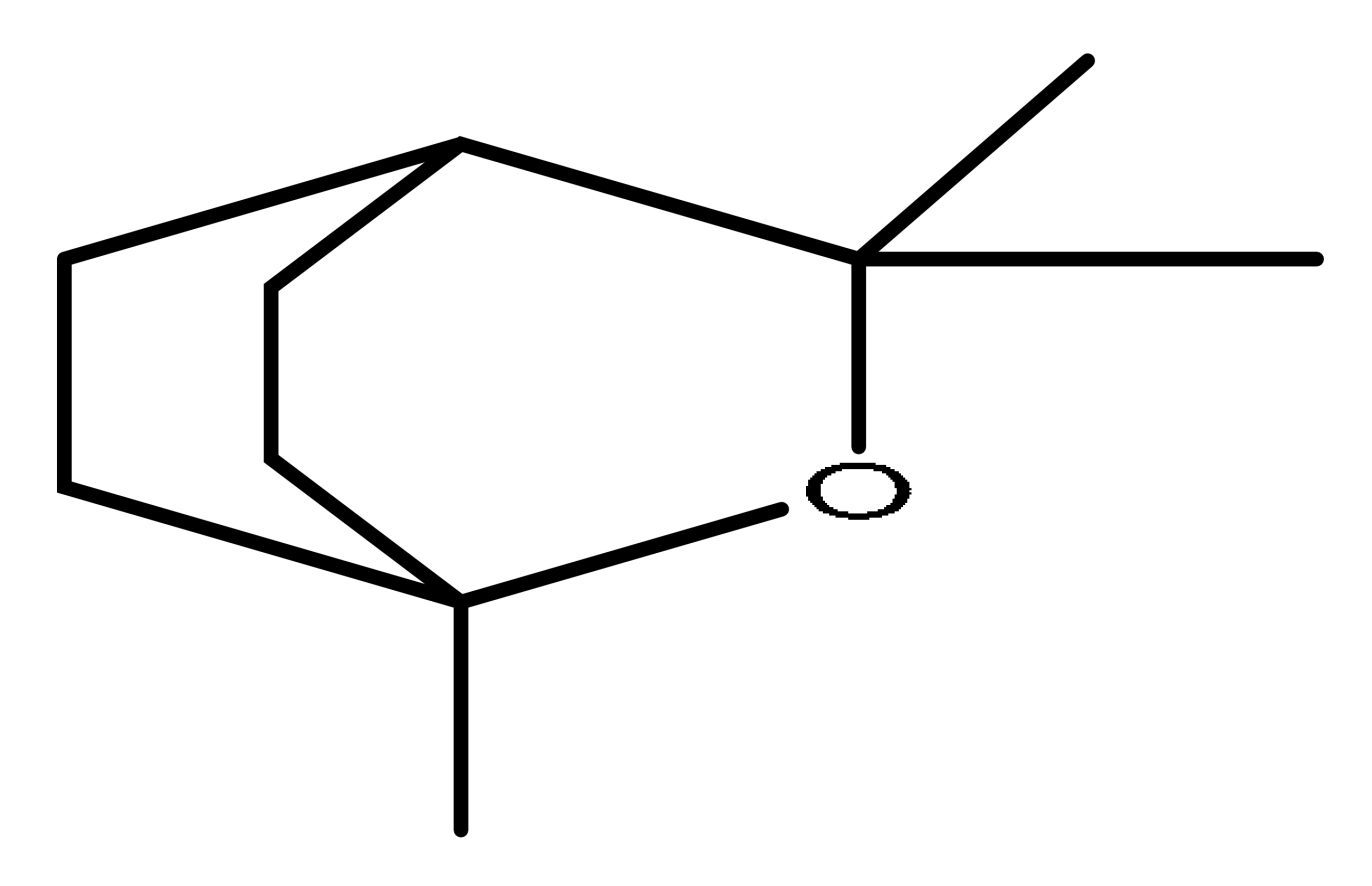	Oxygenated monoterpene	1028	1031	3
3	D-Limonene	4.359	0.98	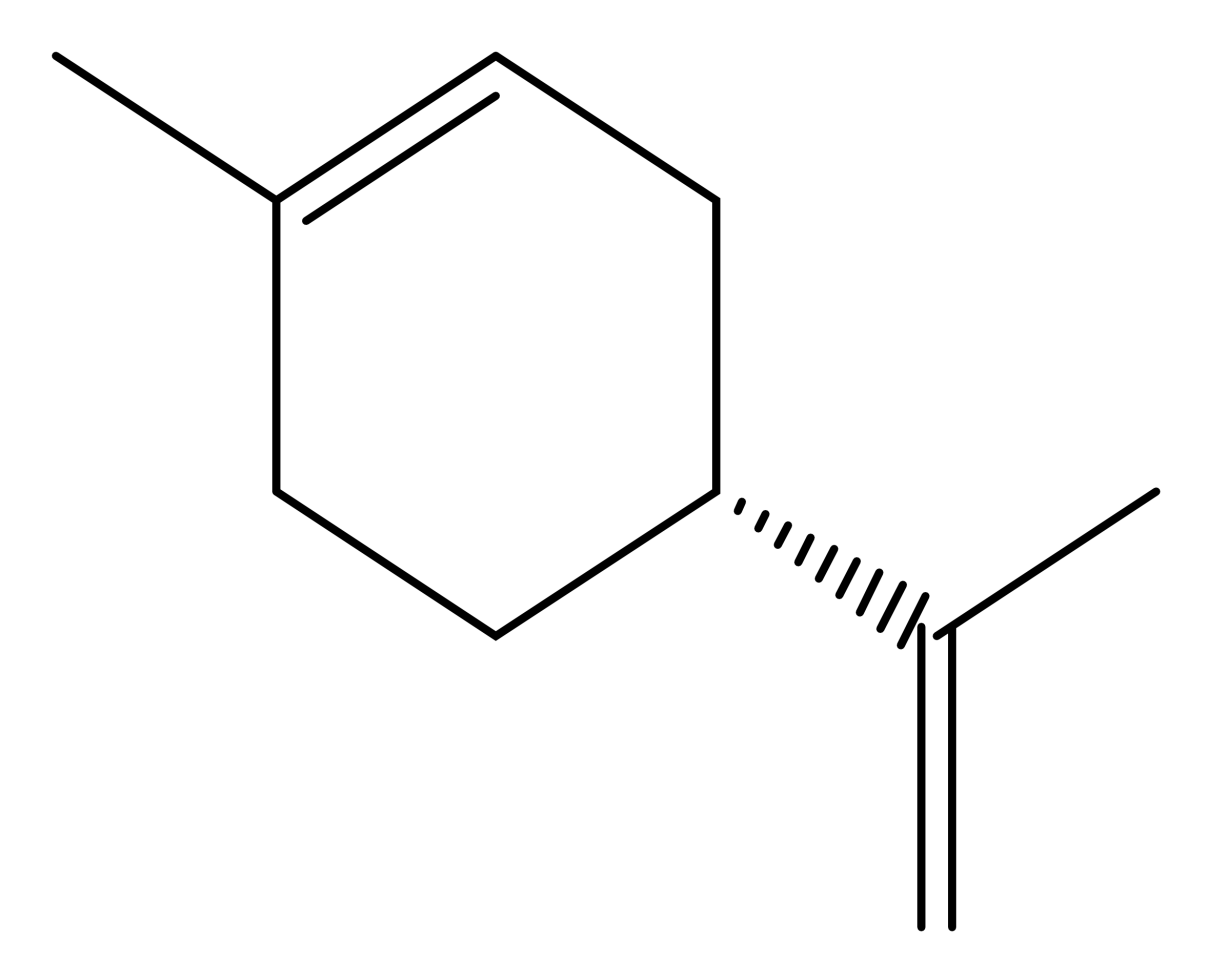	Monoterpene hydrocarbon	1027	1029	2
4	*β*-Linalool	5.325	0.77	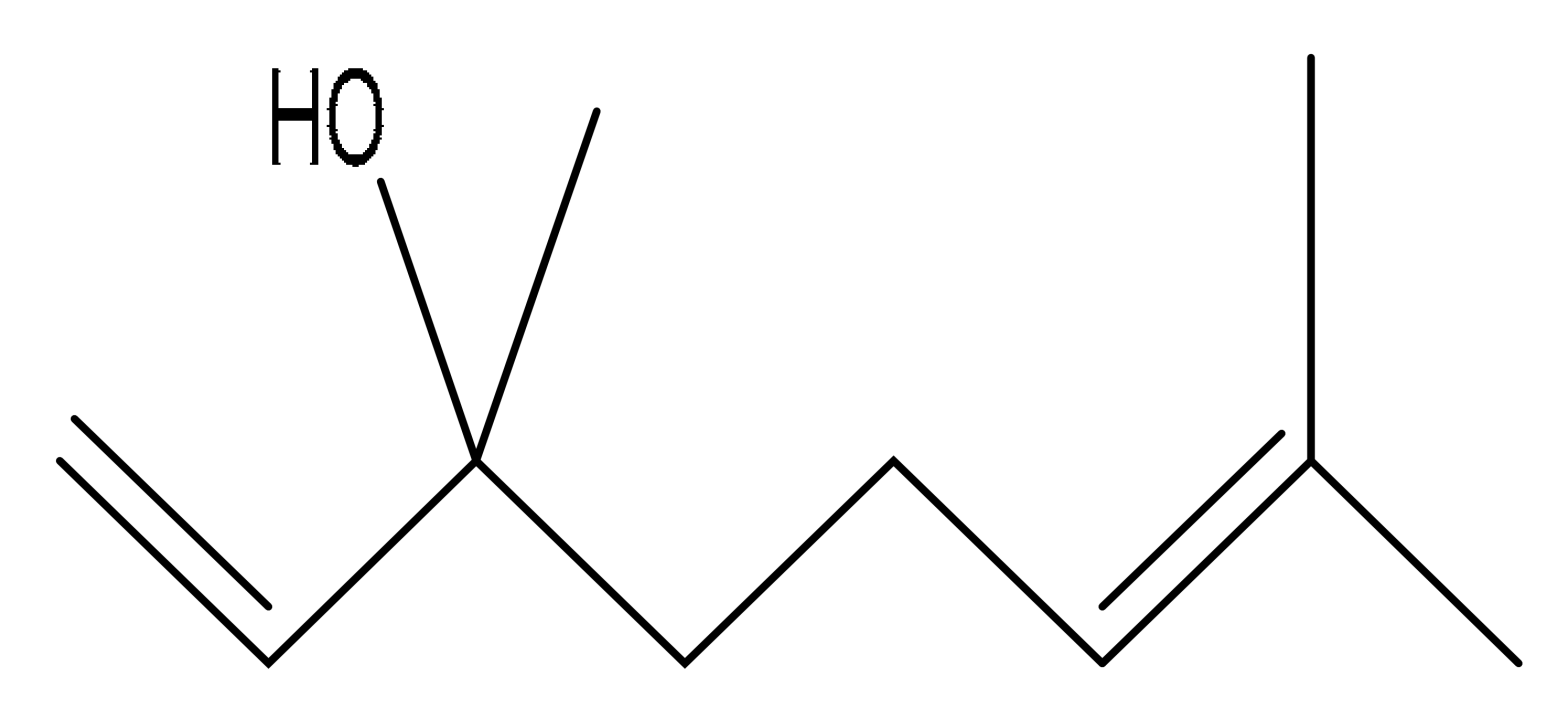	Oxygenated monoterpene	1094	1098	4
5	2-Fenchanol	5.472	0.76	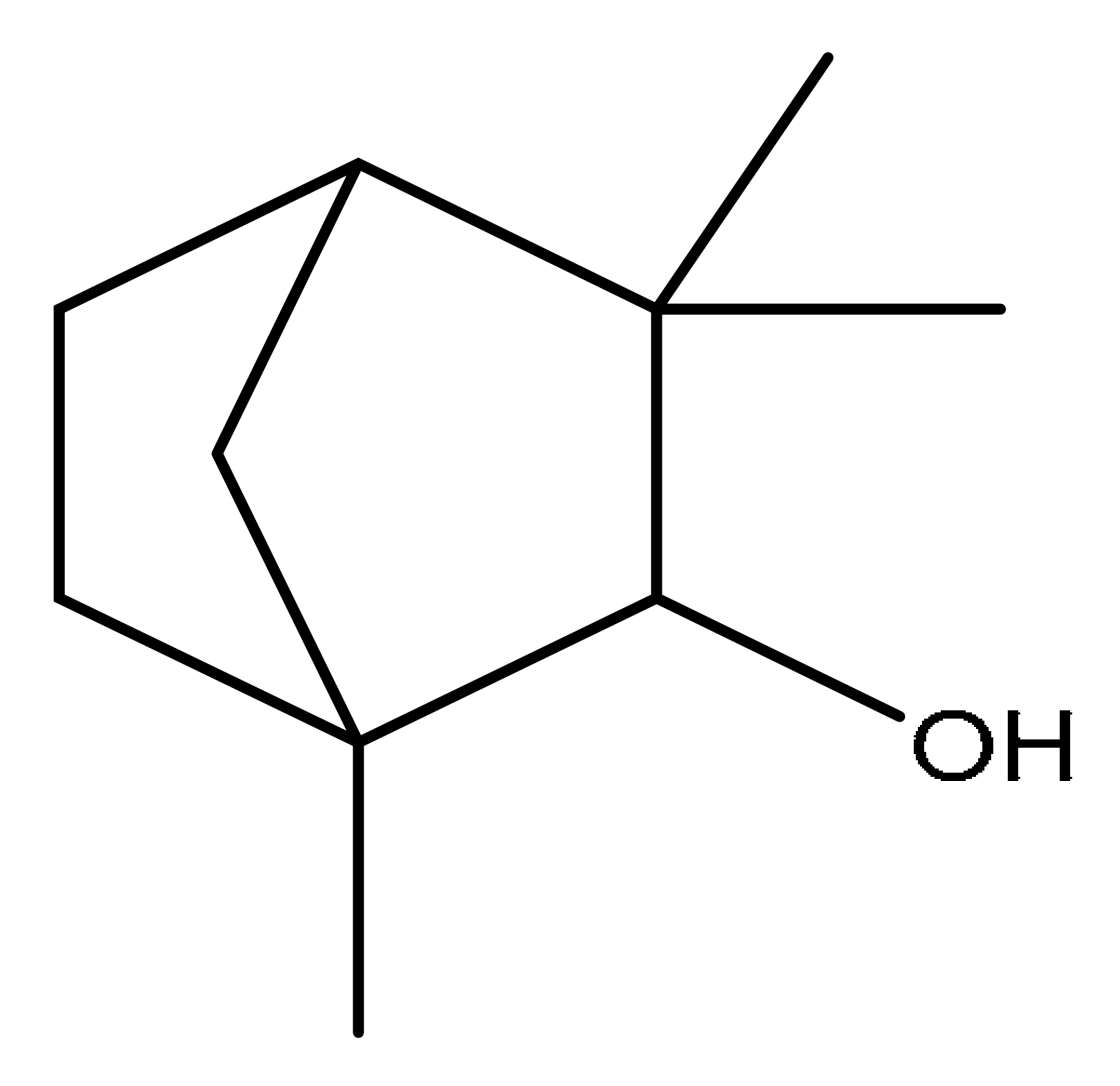	Oxygenated monoterpene	1109	1112	3
6	α-Pinene epoxide	5.566	0.53	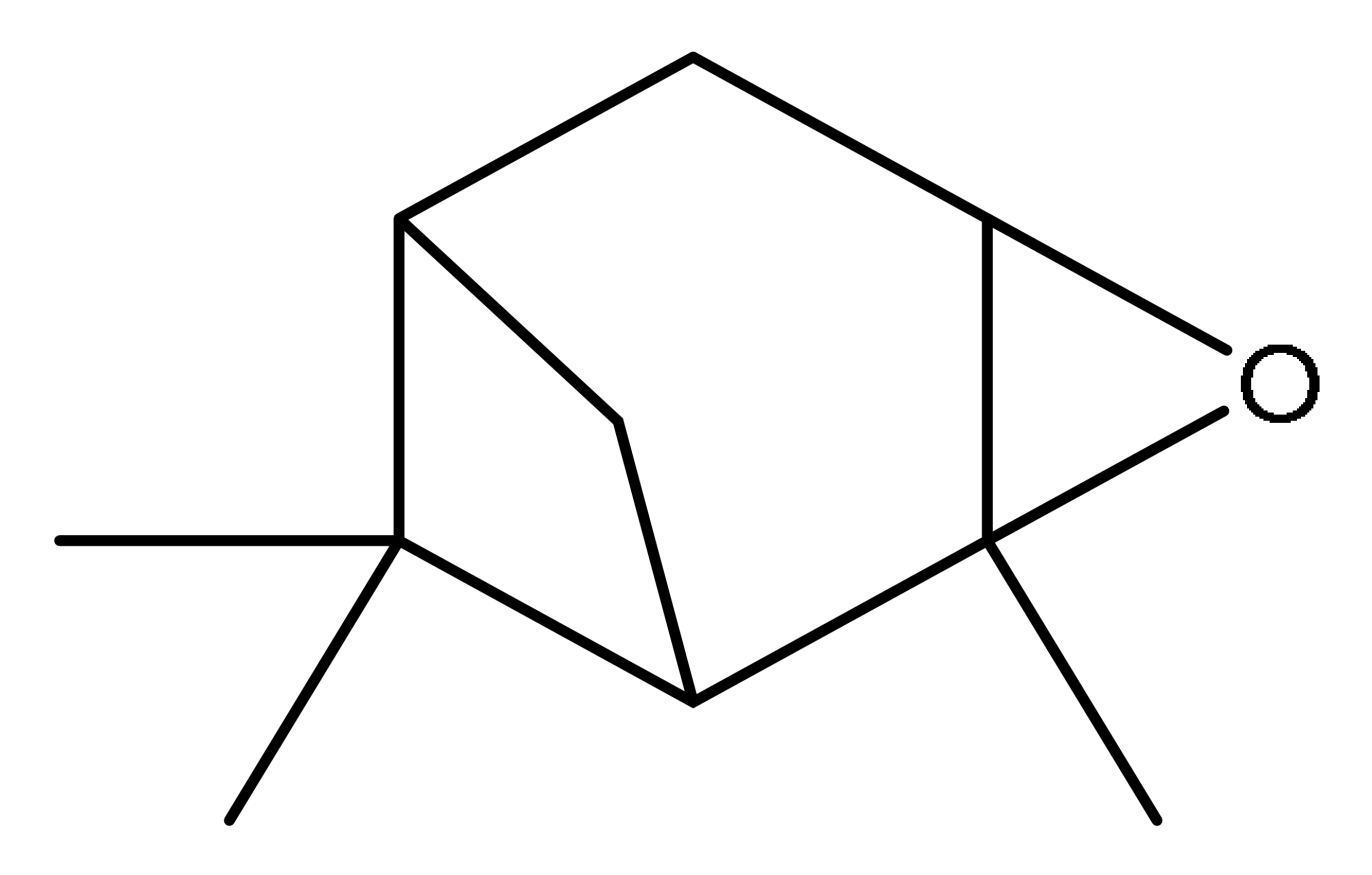	Oxygenated monoterpene	1124	1128	4
7	Camphor	5.771	0.35	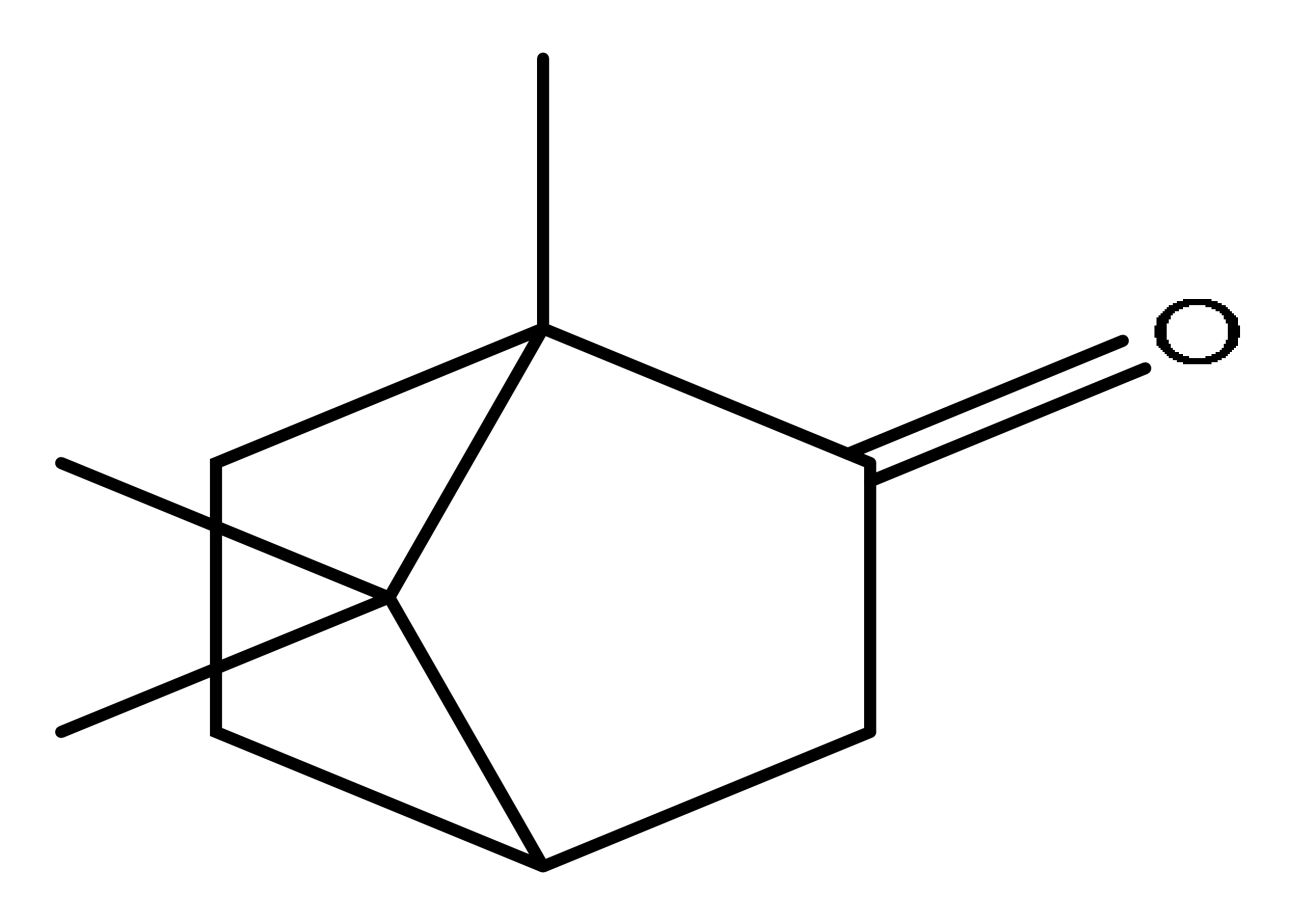	Oxygenated monoterpene	1140	1143	3
8	Trans-Pinocarveol	5.821	0.45	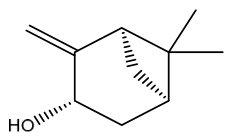	Oxygenated monoterpene	1137	1139	2
9	Cis-Verbenol	5.936	0.29	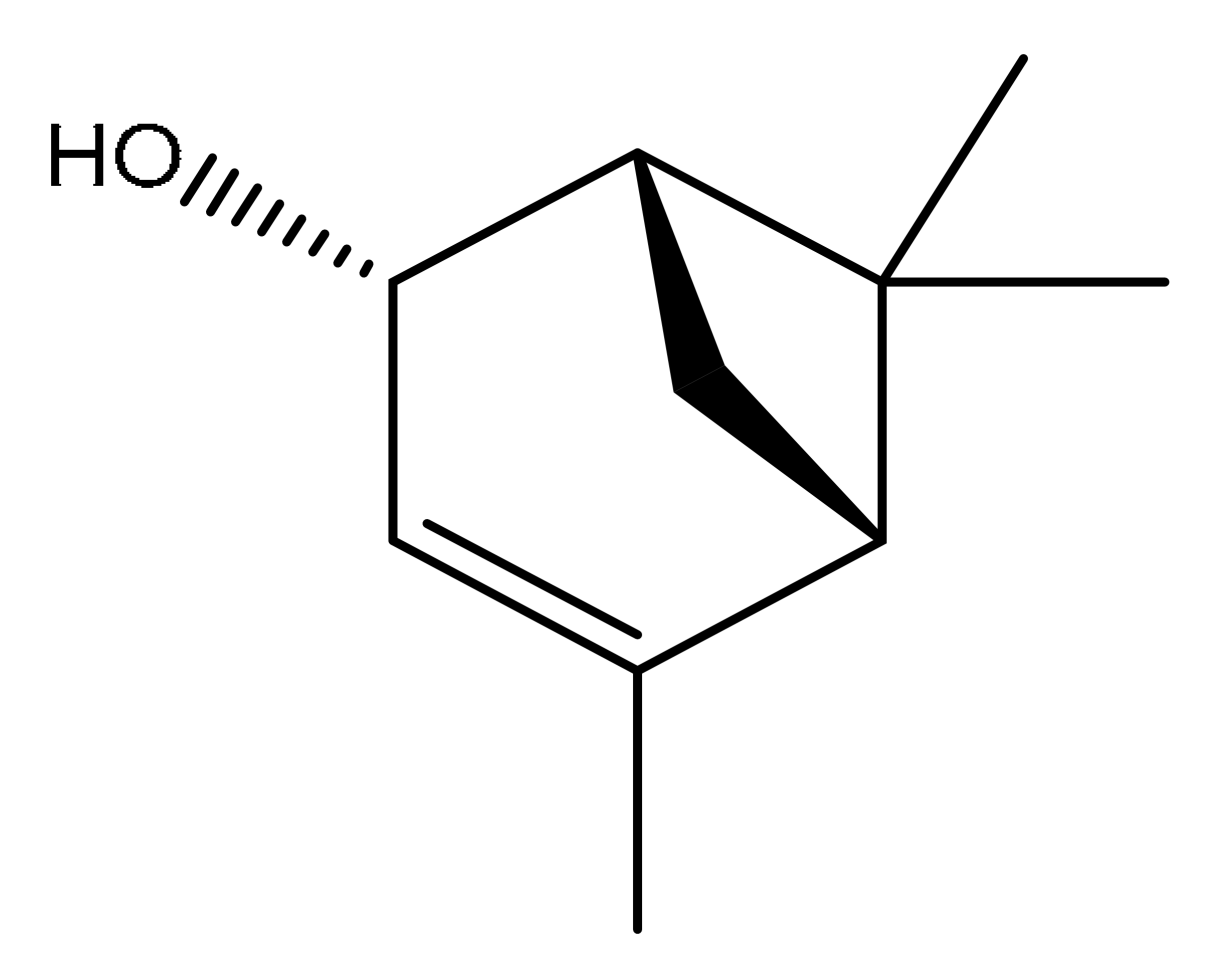	Oxygenated monoterpene	1140	1141	1
10	Camphol	6.209	0.74	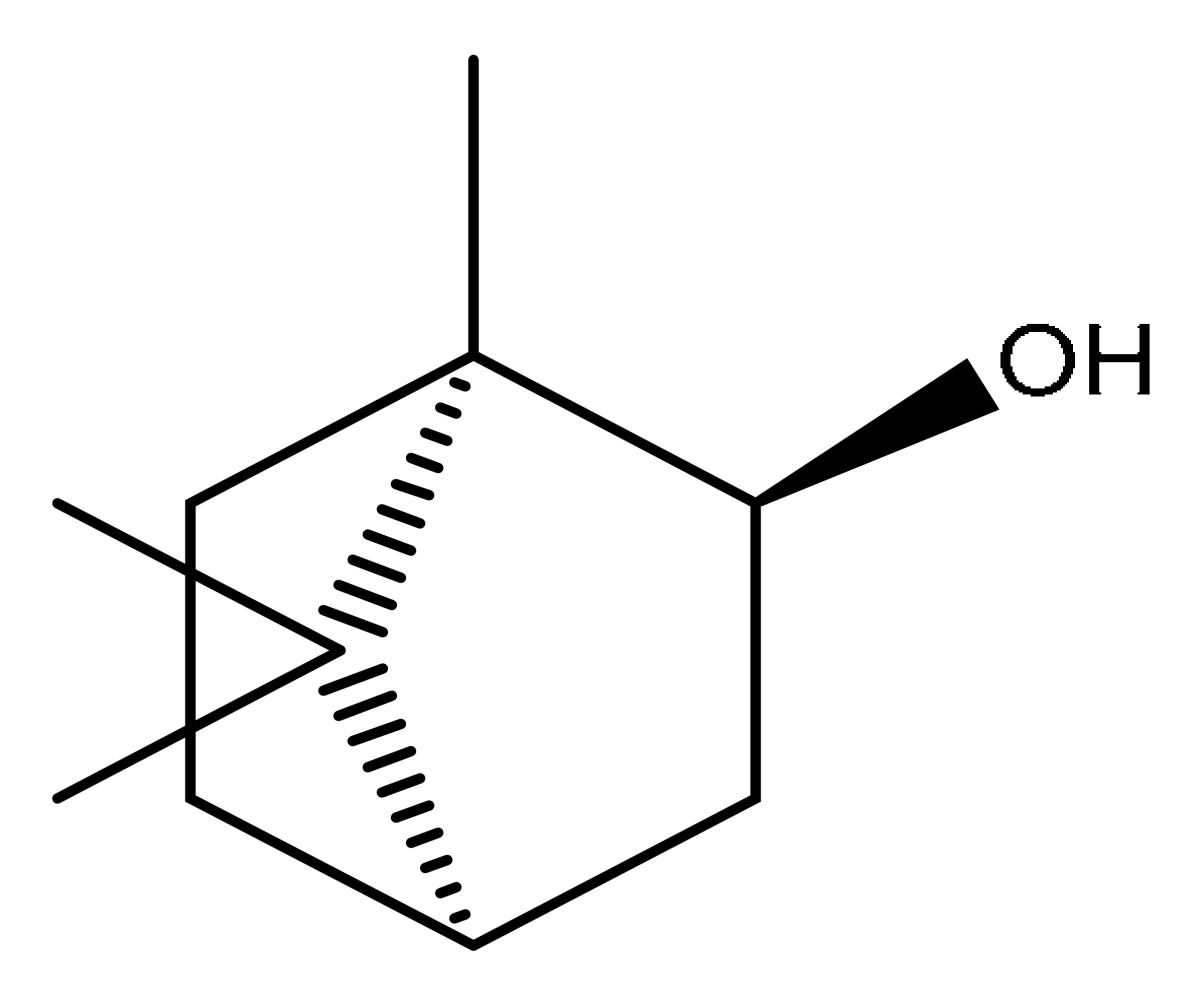	Oxygenated monoterpene	1162	1165	3
11	p-Menth-1-en-4-ol	6.409	0.56	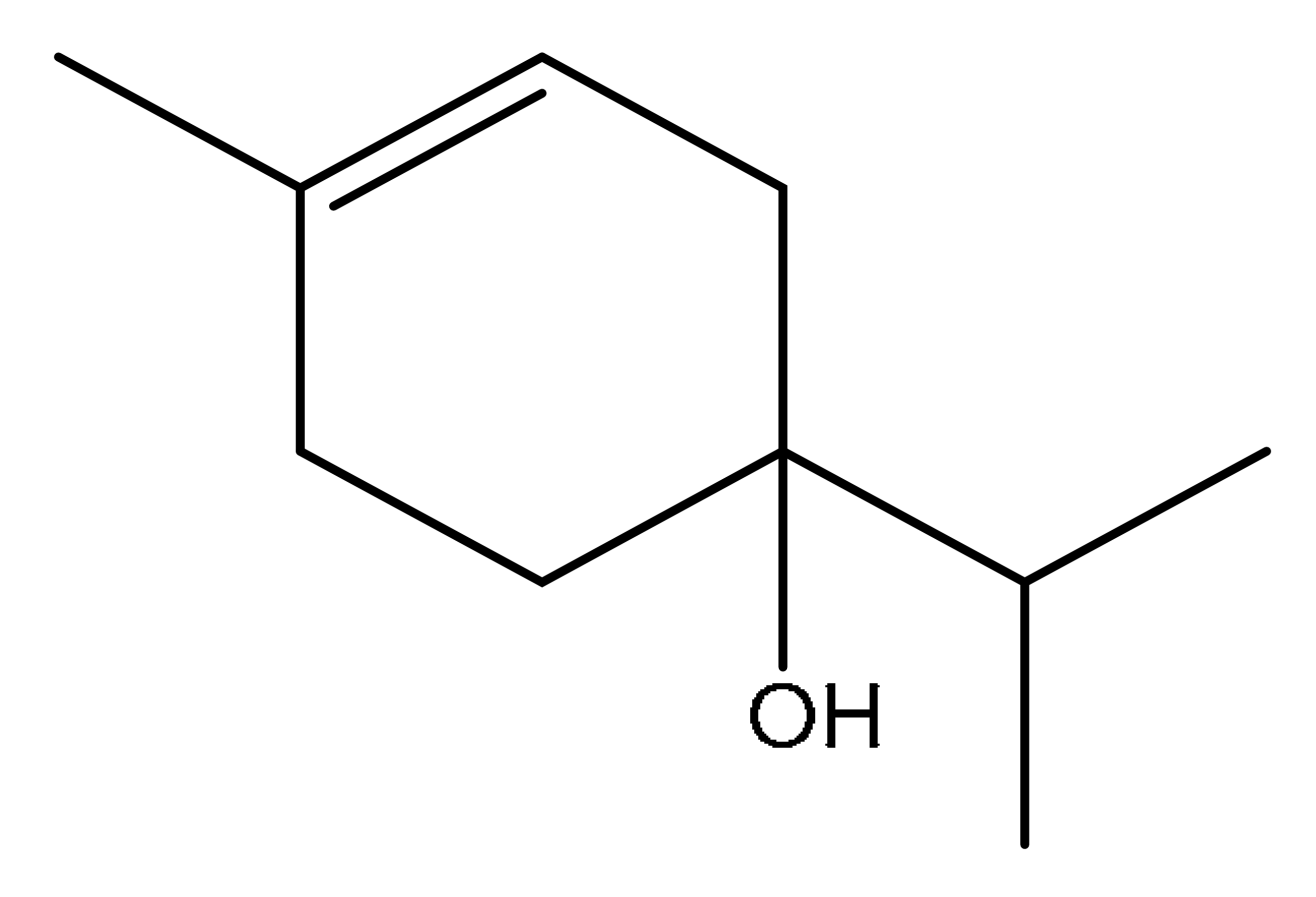	Oxygenated monoterpene	1175	1177	2
12	α-Terpineol	6.574	1.28	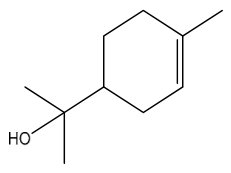	Oxygenated monoterpene	1188	1189	1
13	2-Pinen-4-one	8.513	0.42	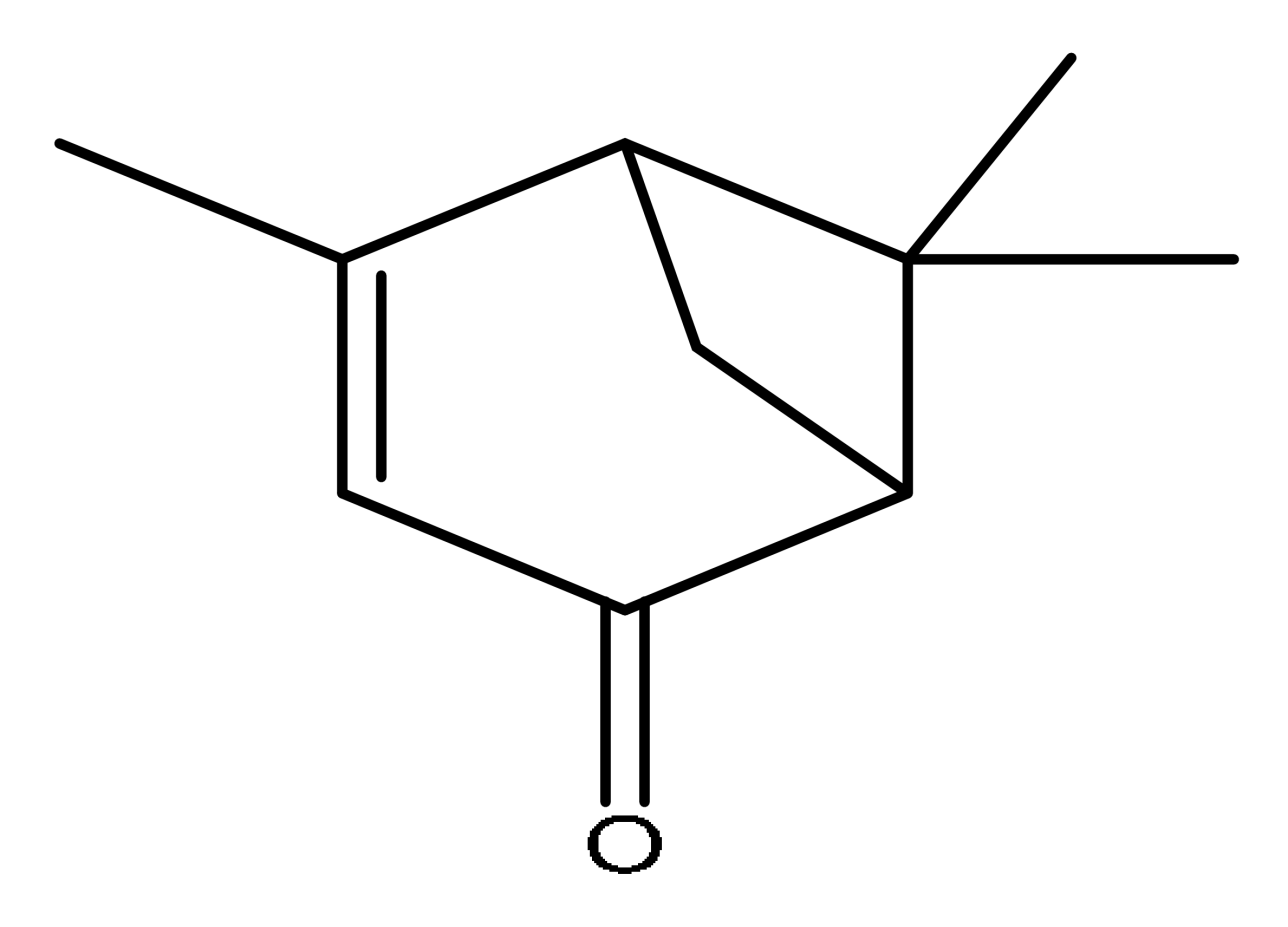	Oxygenated monoterpene	1201	1205	4
14	Caryophyllene	9.974	13.19	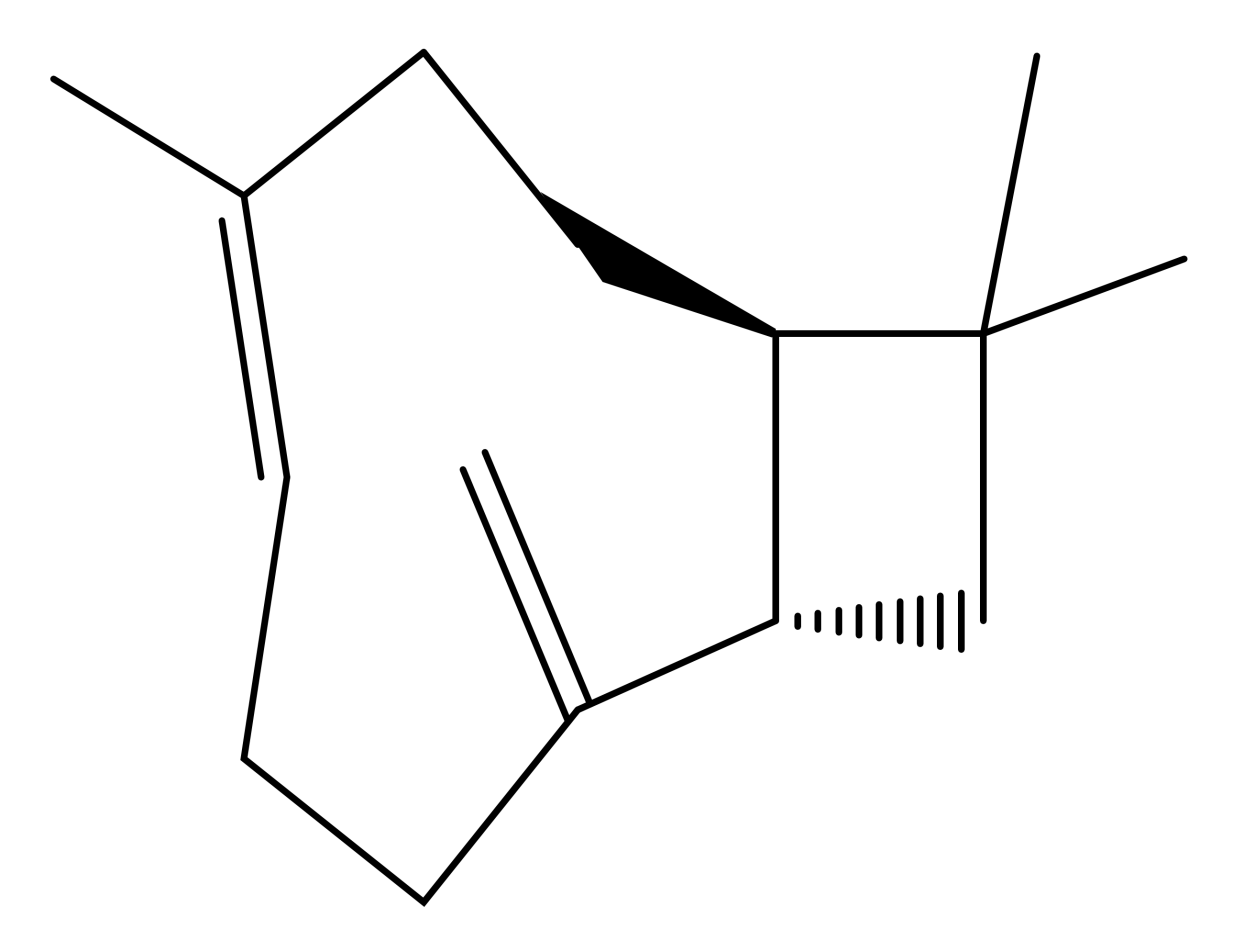	Sesquiterpene hydrocarbon	1417	1418	1
15	α-Bergamotene	10.232	2.51	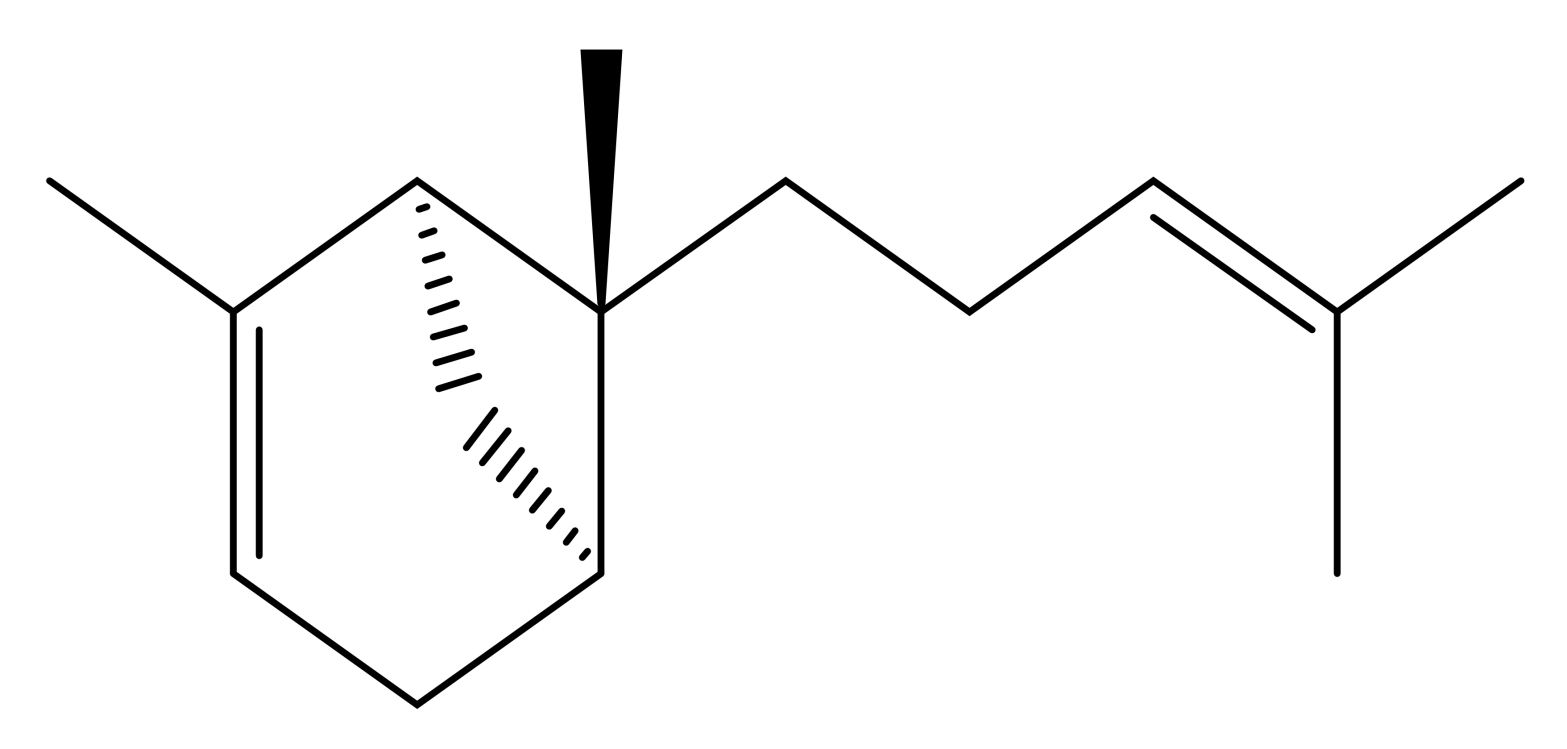	Sesquiterpene hydrocarbon	1432	1435	3
16	Cis-alpha-Bisabolene	10.399	5.21	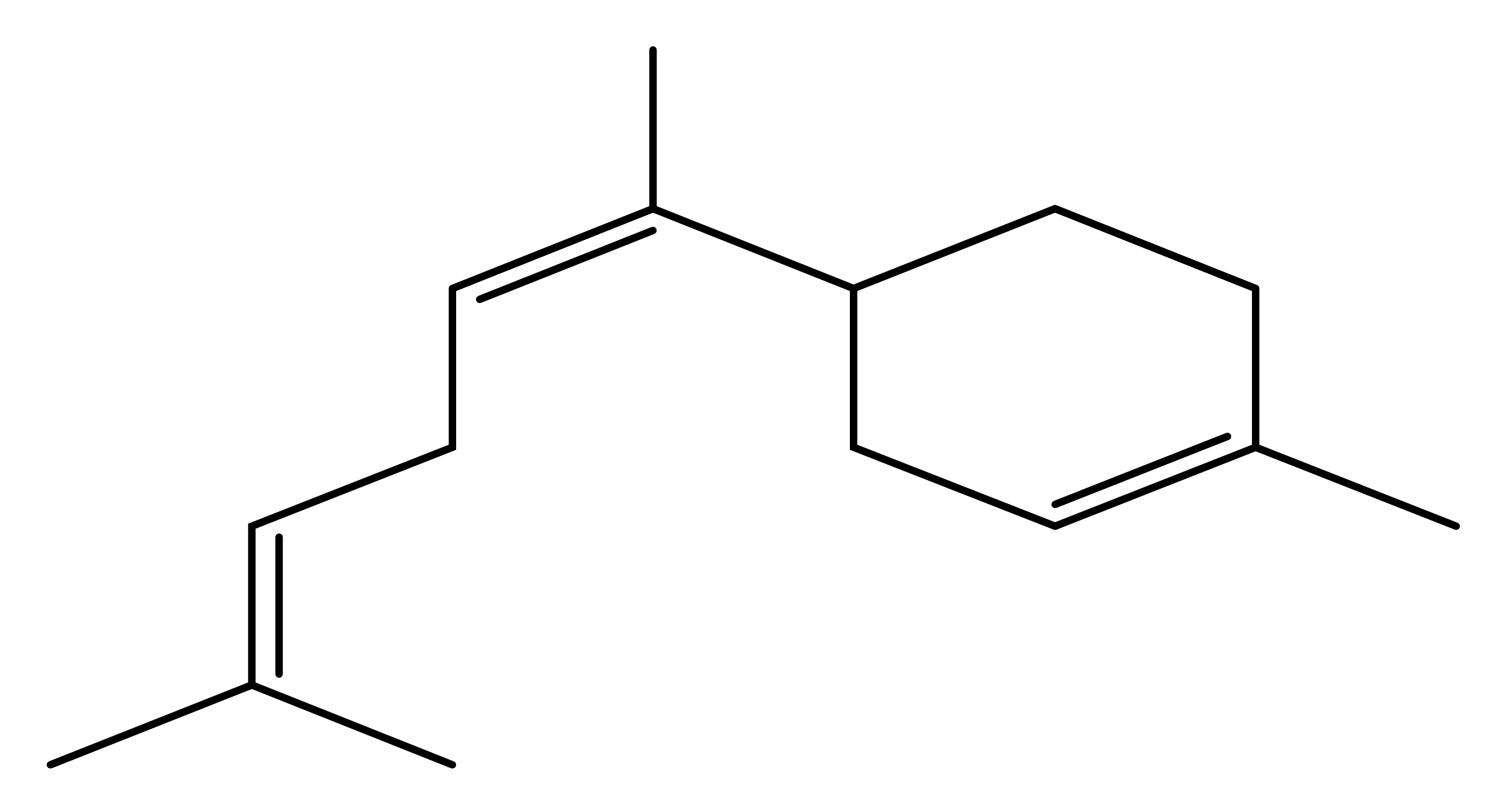	Sesquiterpene hydrocarbon	1500	1504	4
17	*β*-Farnesene	10.467	2.24	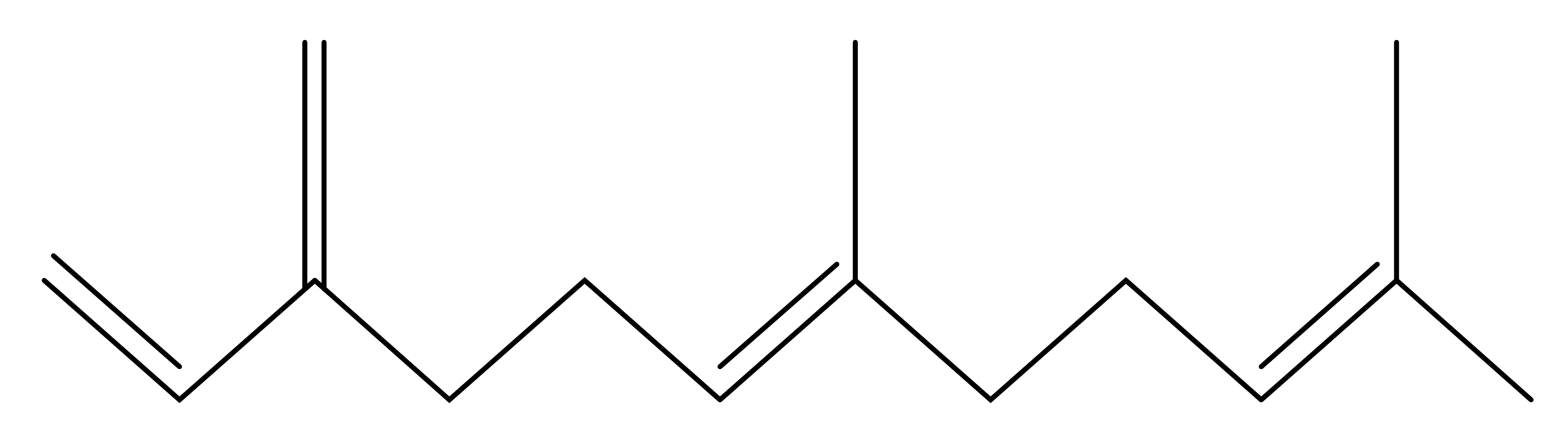	Sesquiterpene hydrocarbon	1456	1458	2
18	*β*-Eudesmene	10.802	2.87	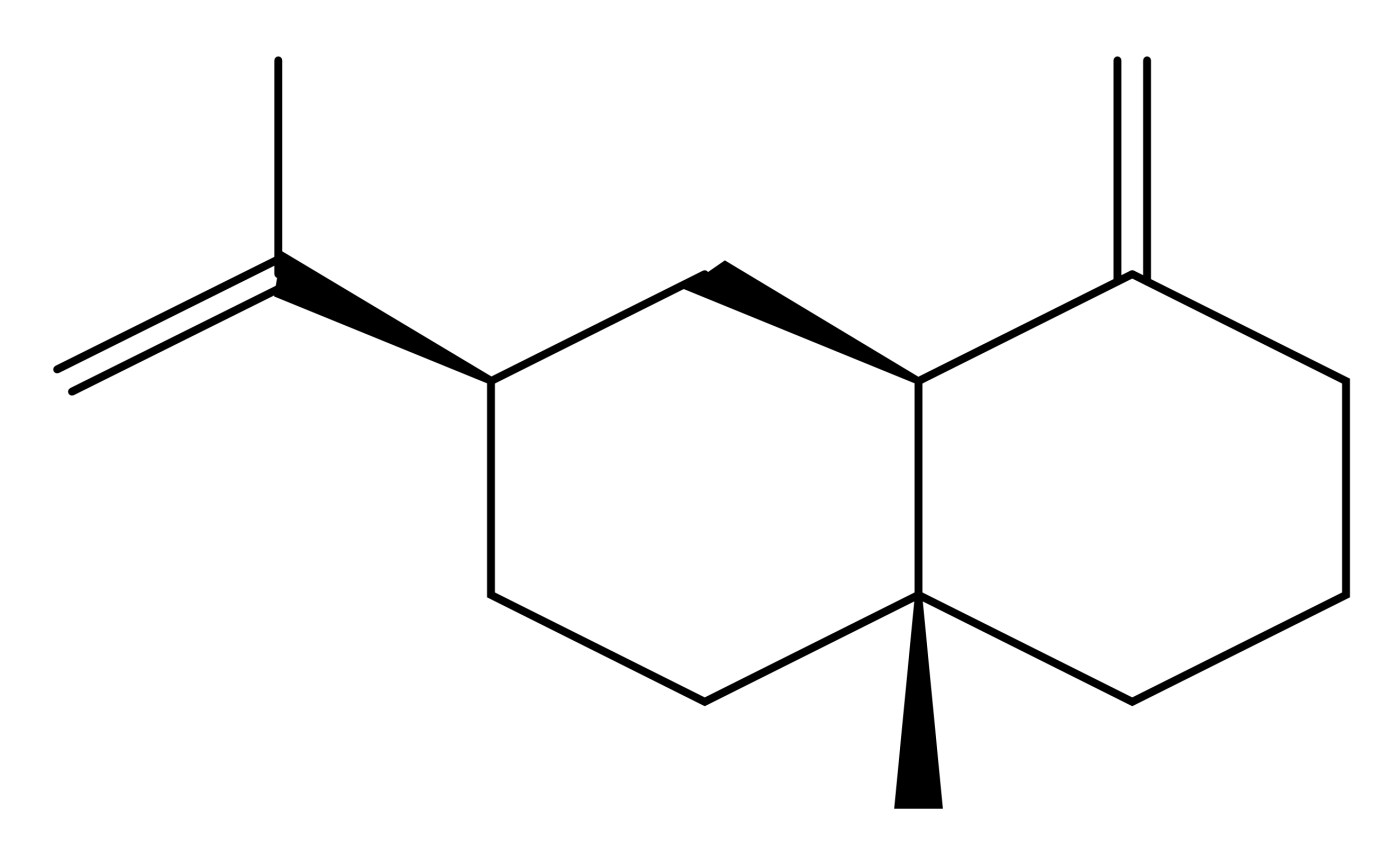	Sesquiterpene hydrocarbon	1491	1494	3
19	γ-Gurjunene	10.933	2.53	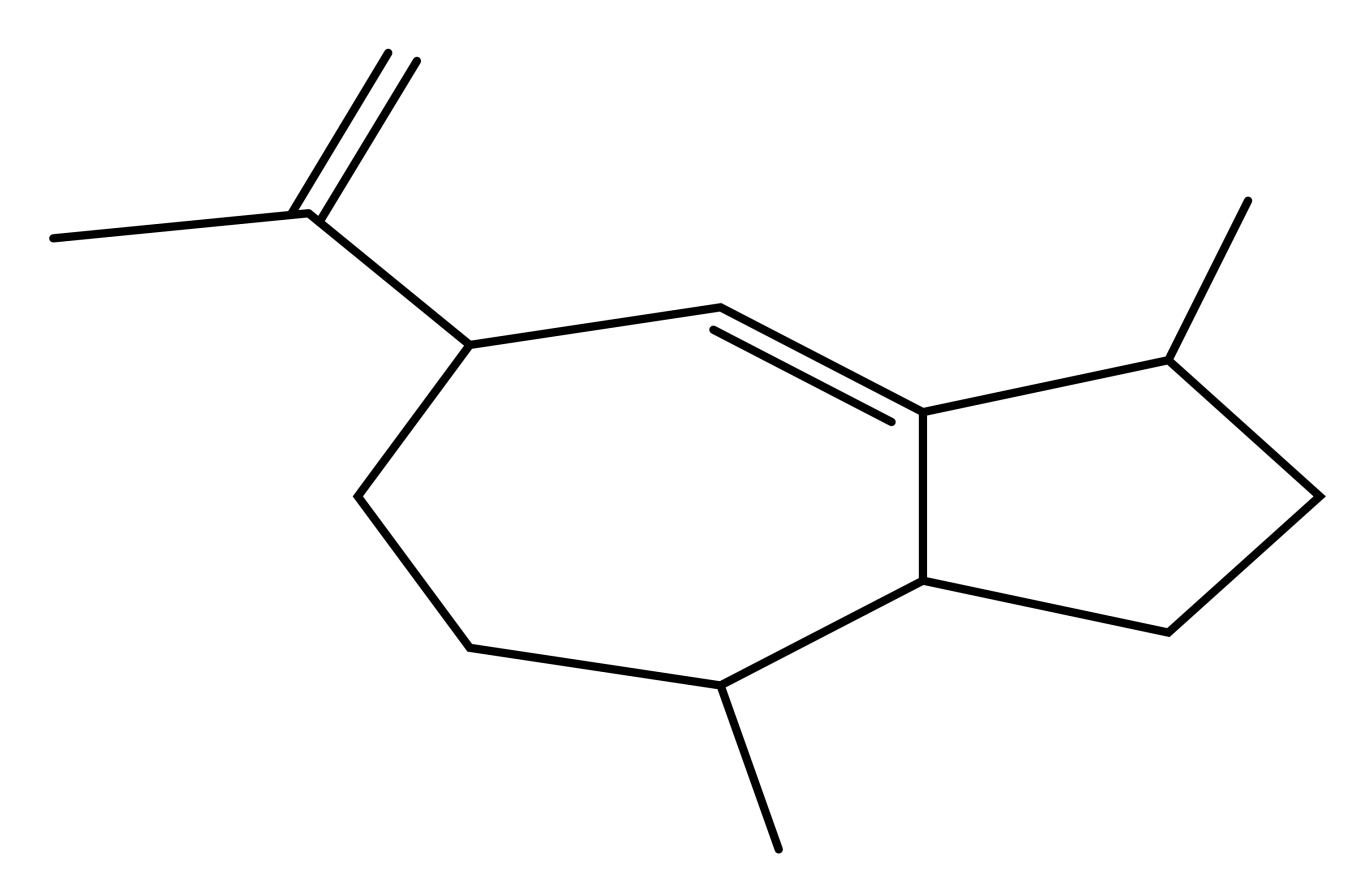	Sesquiterpene hydrocarbon	1475	1477	2
20	α-Bulnesene	11.067	1.13	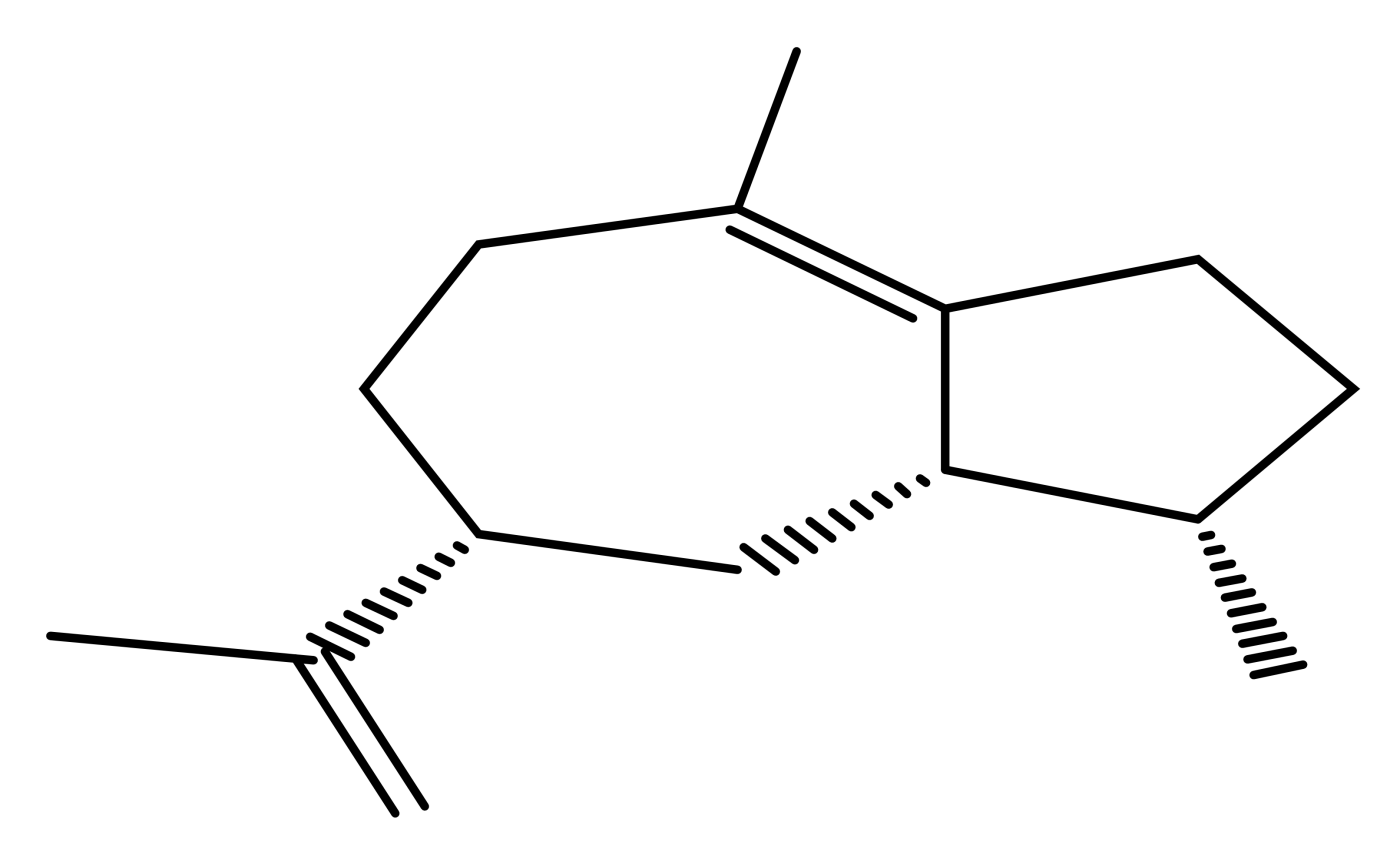	Sesquiterpene hydrocarbon	1503	1505	2
21	Isocaryophillene	11.131	1.54	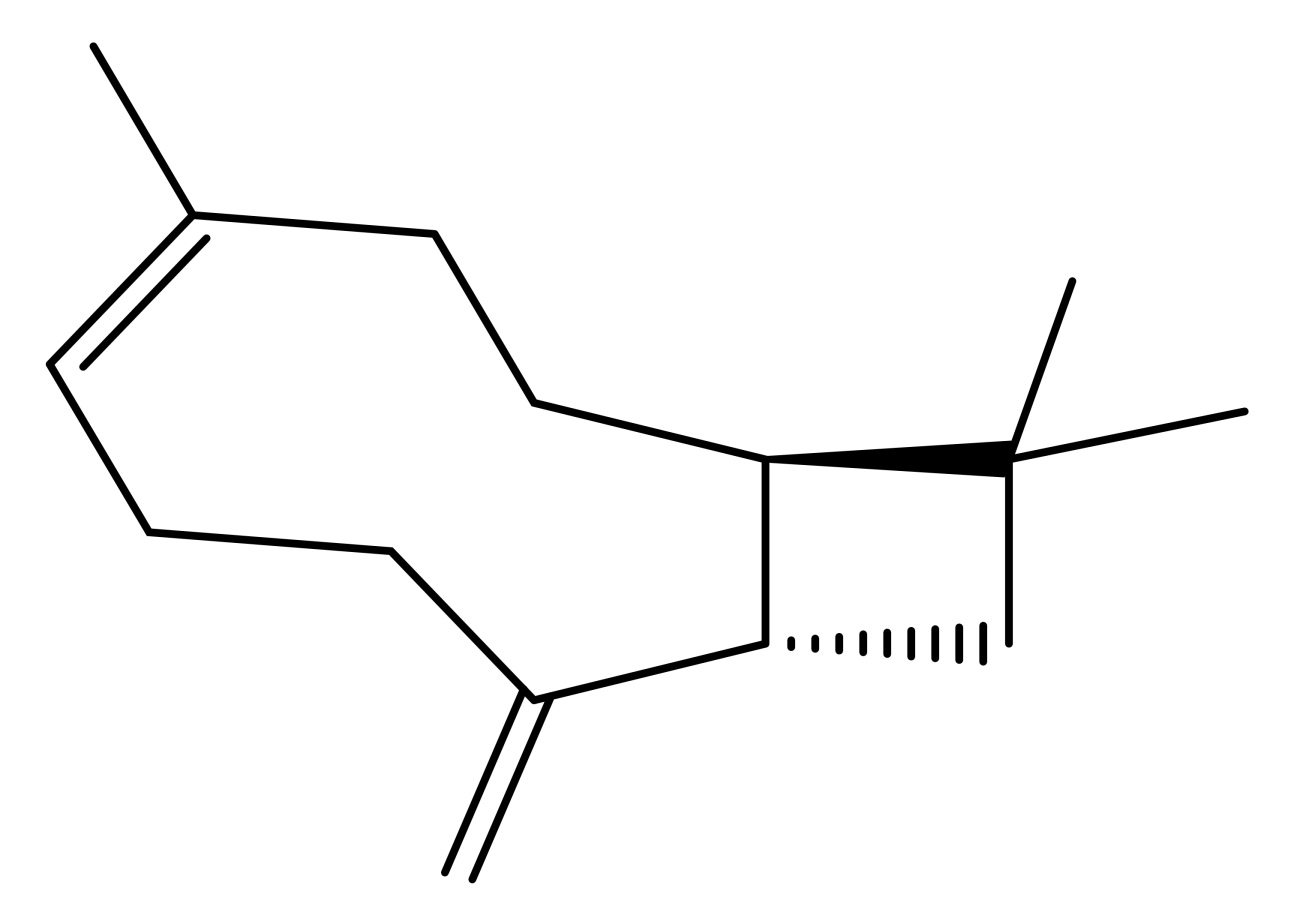	Sesquiterpene hydrocarbon	1402	1405	3
22	α-Gurjunene	11.258	2.43	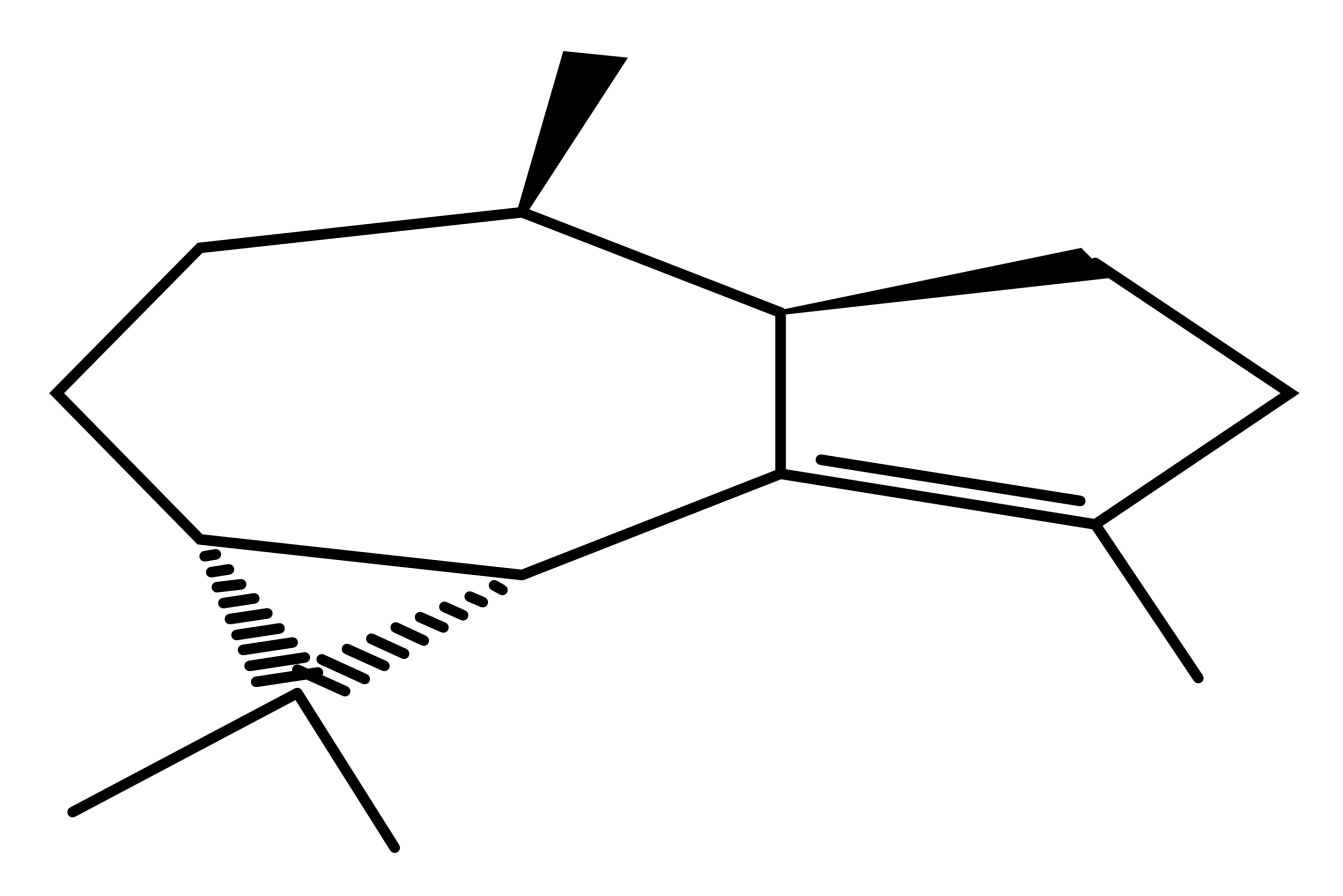	Sesquiterpene hydrocarbon	1406	1409	3
23	Valencene	11.409	5.80	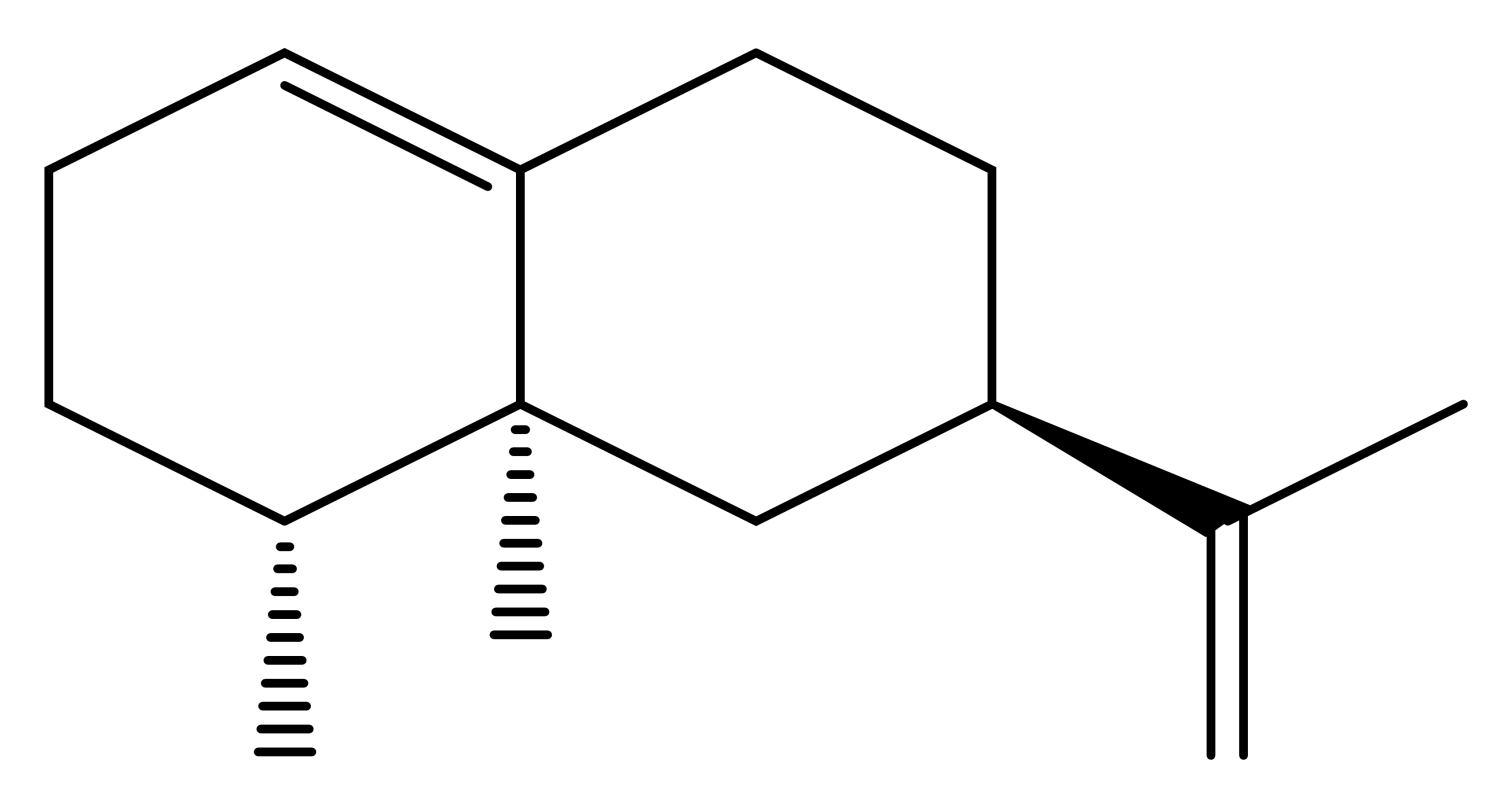	Sesquiterpene hydrocarbon	1492	1496	4
24	Selina-3,7(11)-diene	11.521	8.00	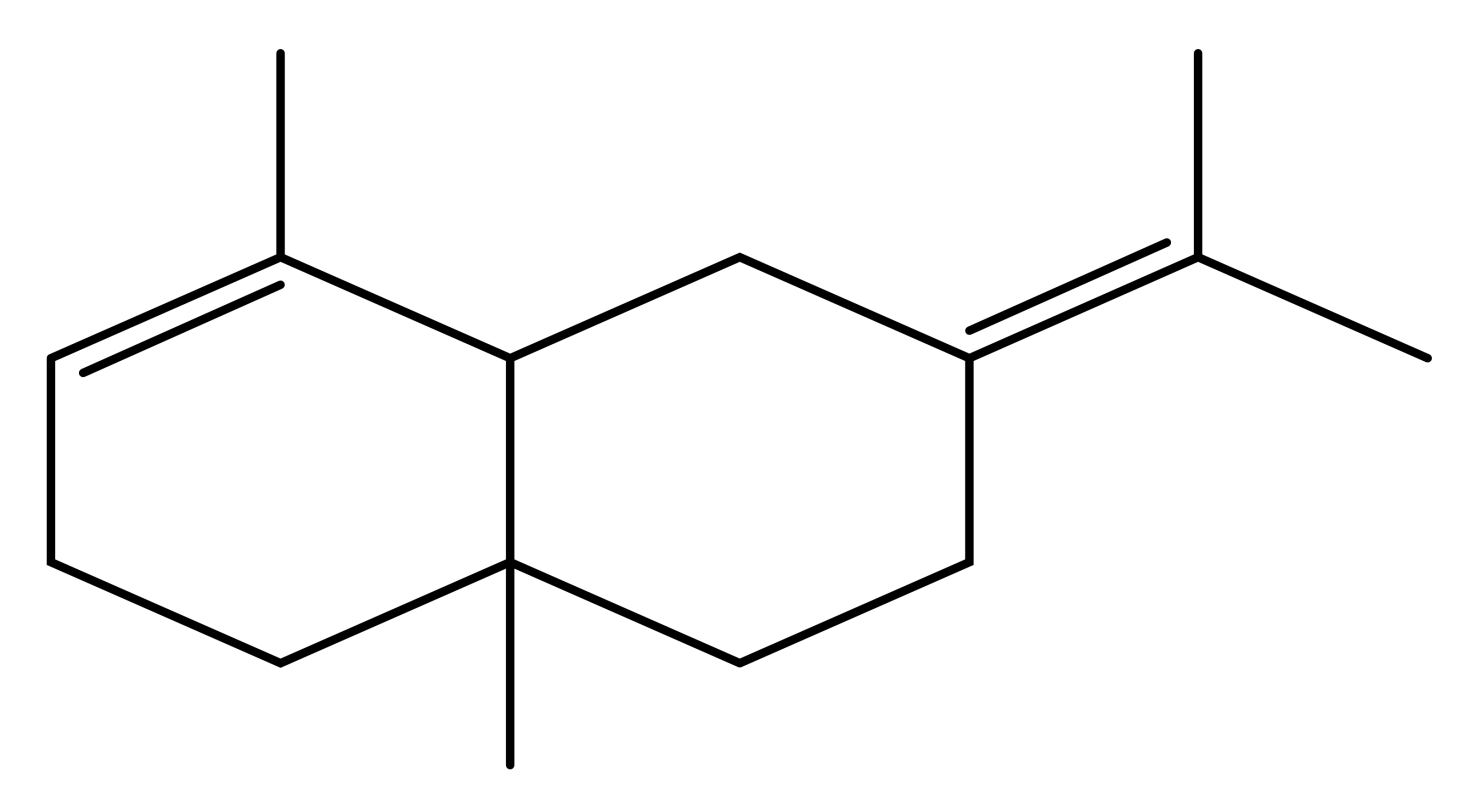	Sesquiterpene hydrocarbon	1529	1532	3
25	α-Farnesene	11.721	2.33	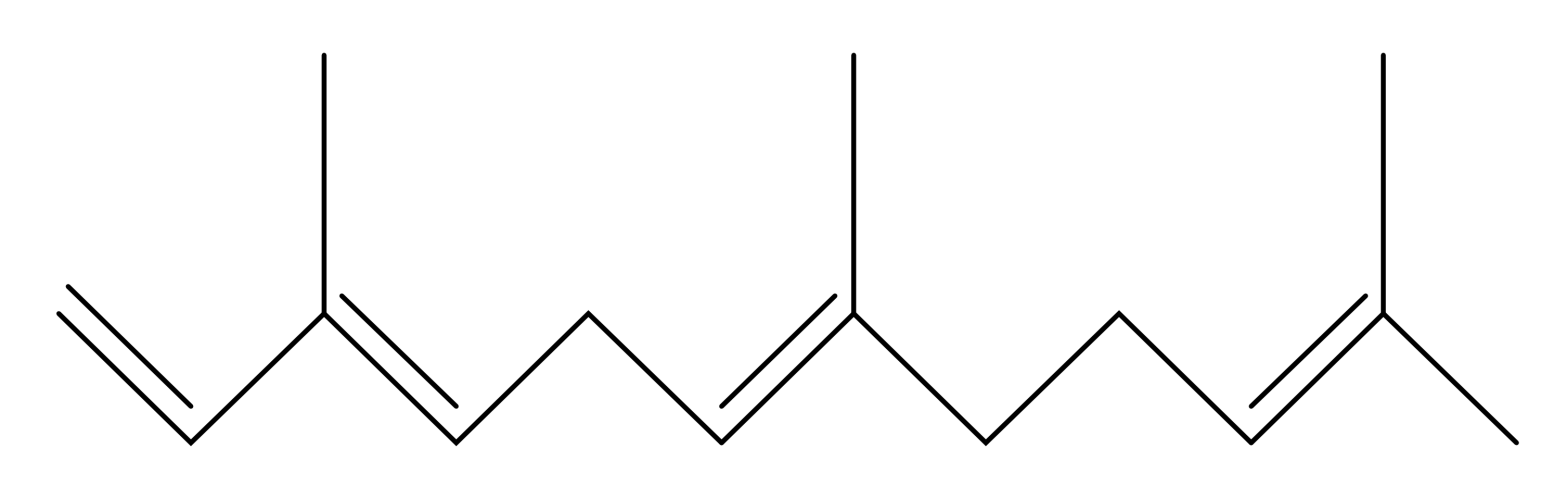	Sesquiterpene hydrocarbon	1503	1506	3
26	Isoaromadendrene Epoxide	11.883	3.55	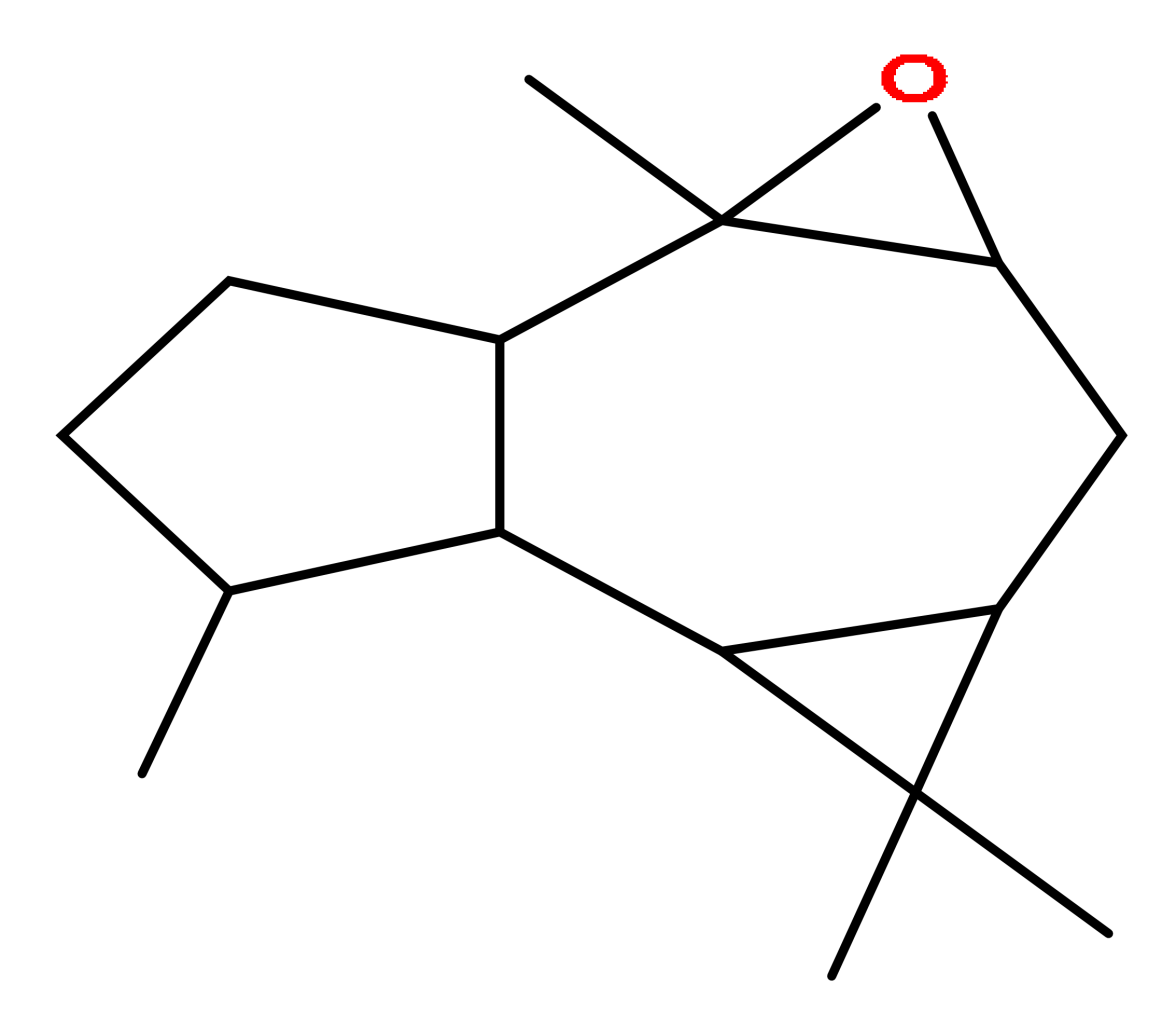	Oxygenated sesquiterpene	1528	1533	5
27	Guaiol	12.107	5.58	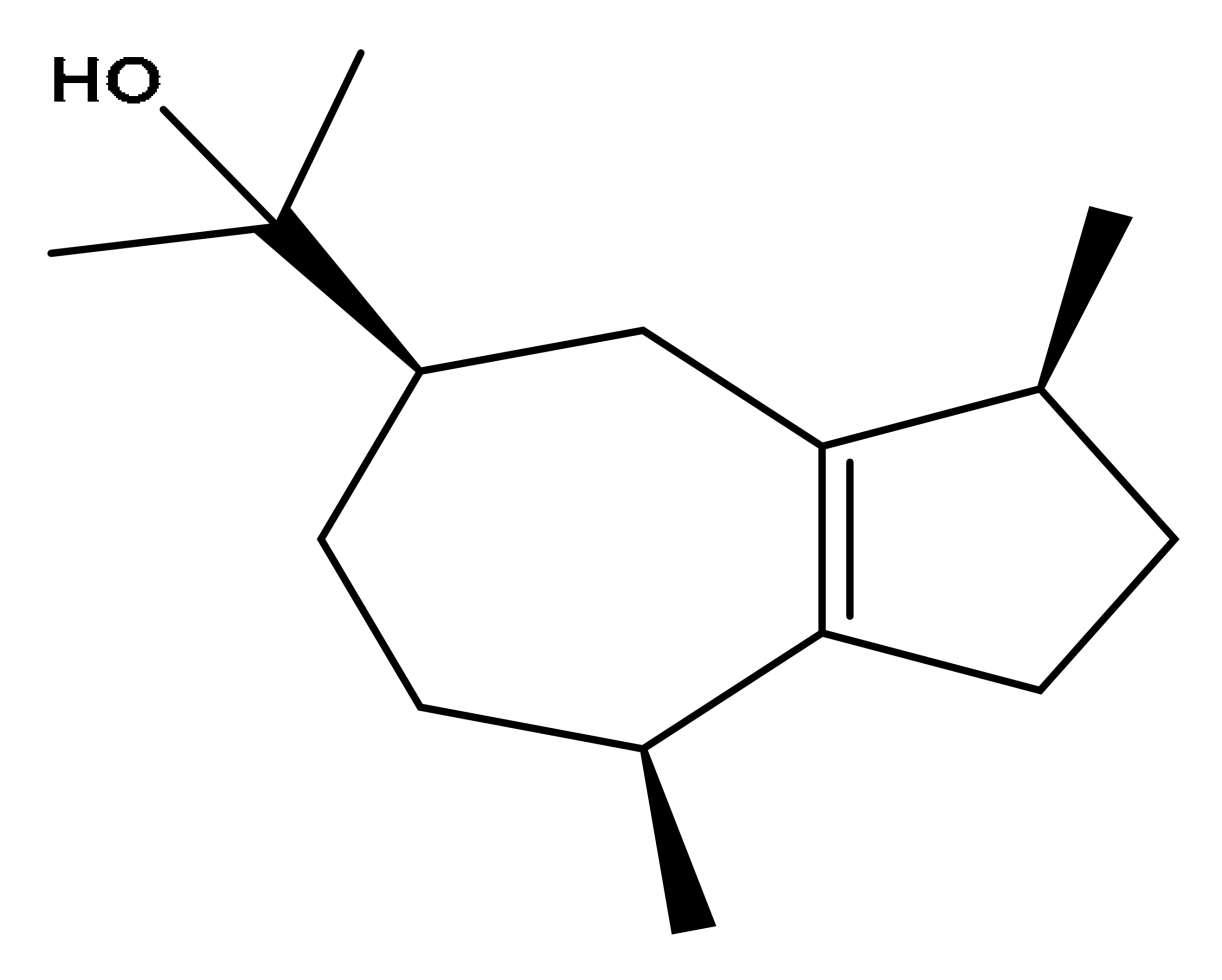	Oxygenated sesquiterpene	1597	1600	3
28	γ-Eudesmol	12.350	6.57	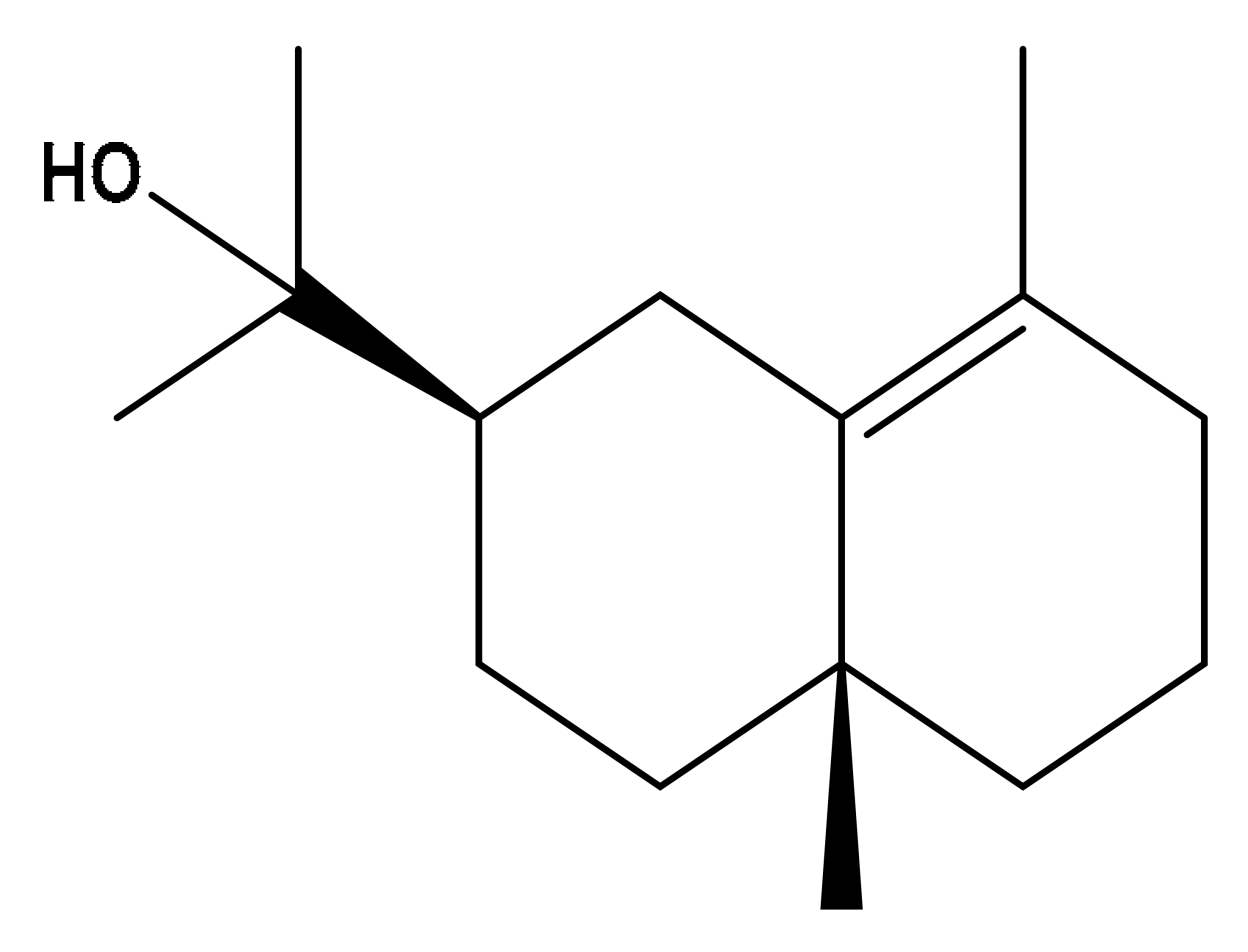	Oxygenated sesquiterpene	1627	1630	3
29	Epiglobulol	12.466	6.04	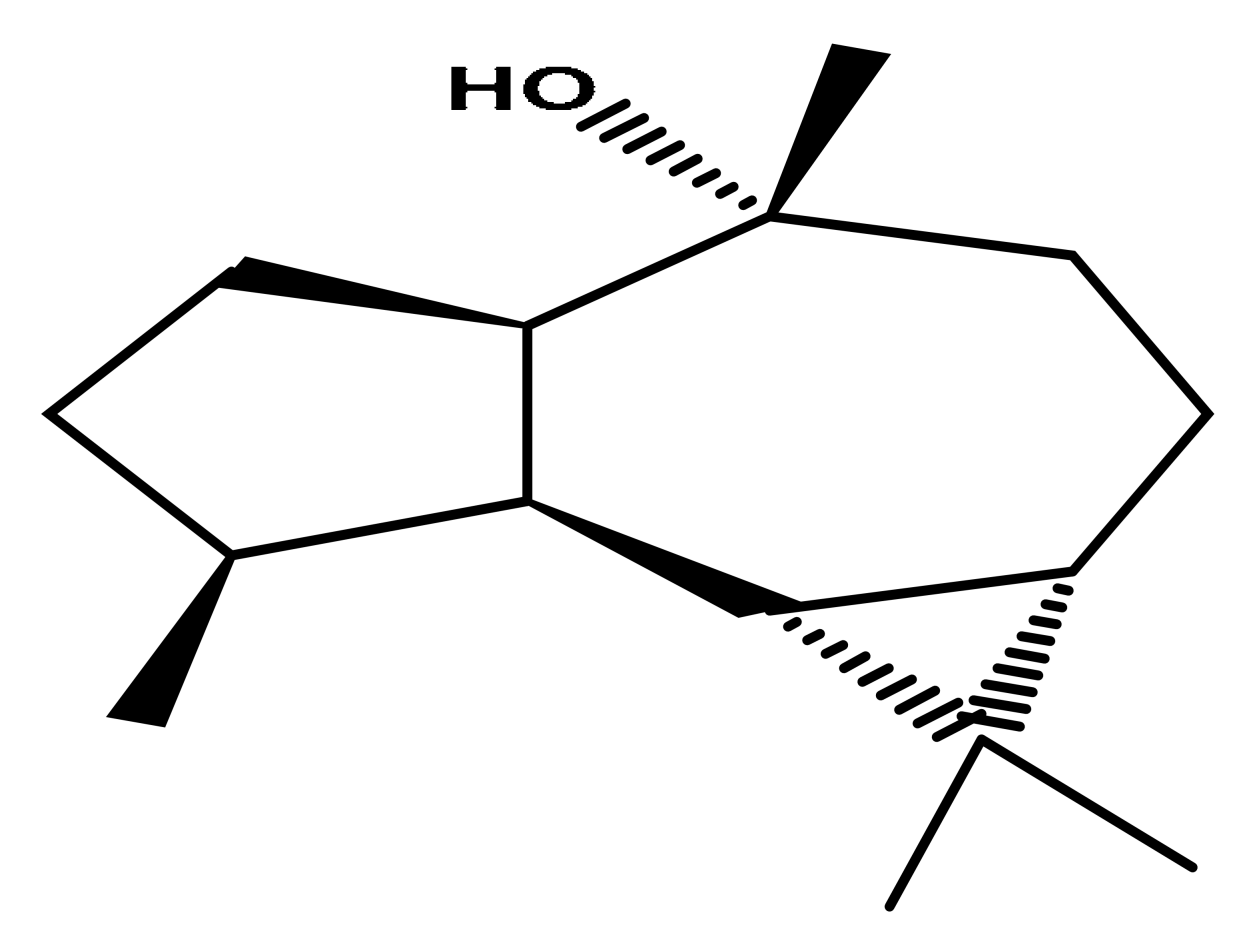	Oxygenated sesquiterpene	1583	1585	2
30	α-Copaen-11-ol	12.587	3.39	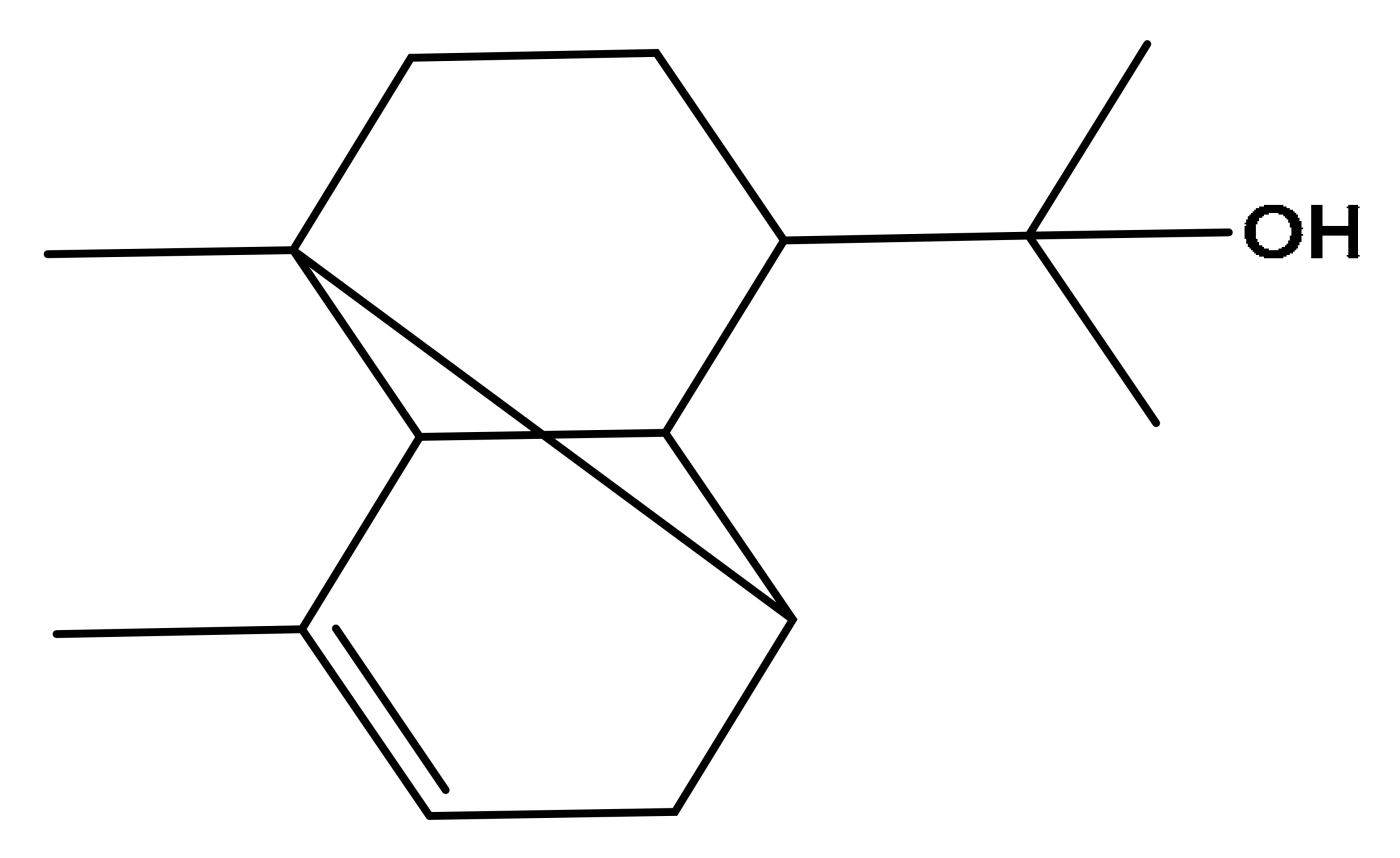	Oxygenated sesquiterpene	1630	1634	4
31	Cubenol	12.738	6.62	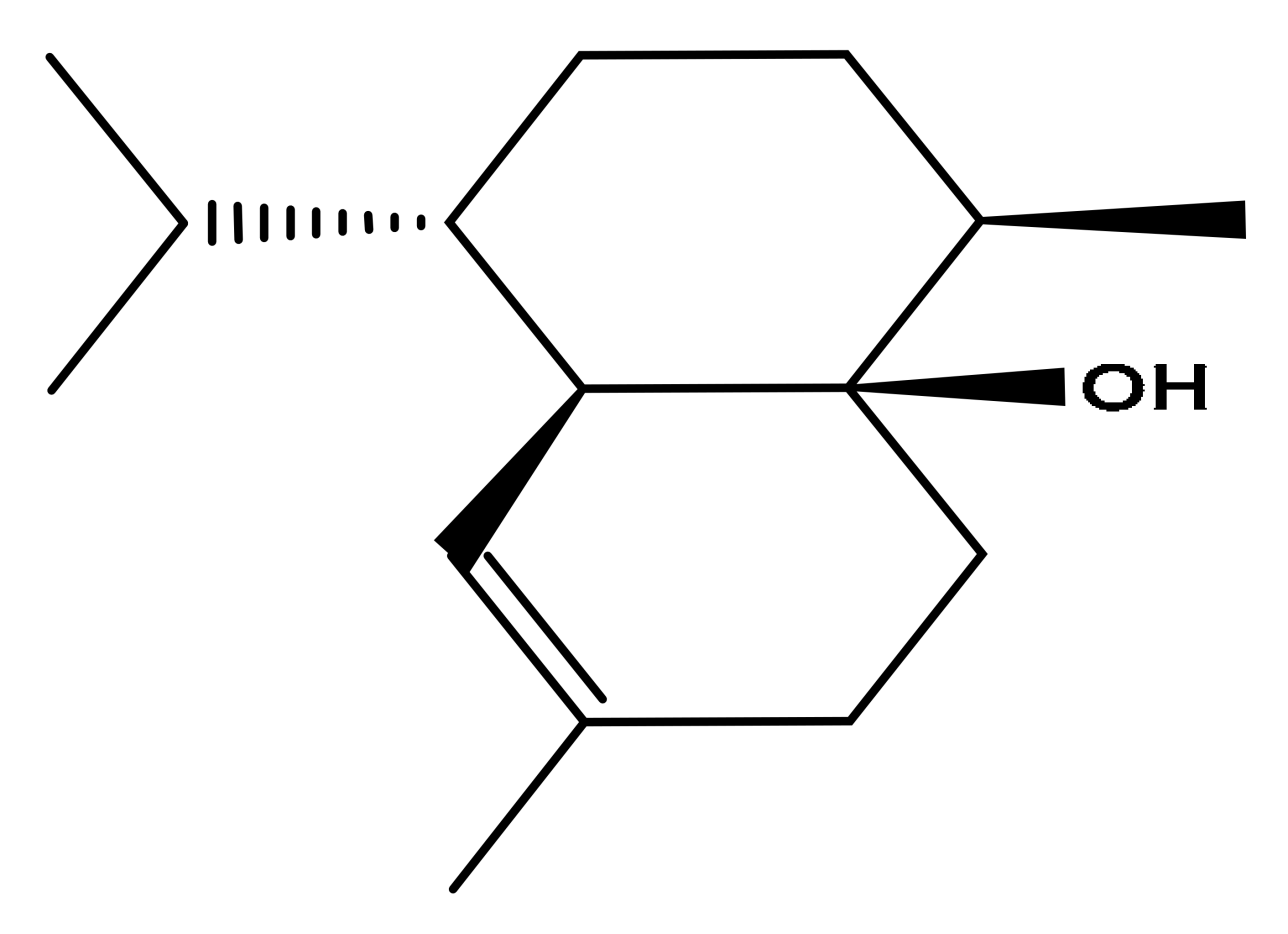	Oxygenated sesquiterpene	1640	1643	3
32	Bulnesol	12.897	5.27	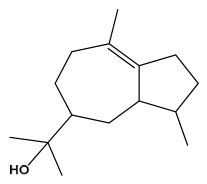	Oxygenated sesquiterpene	1666	1668	2
33	α-Bisabolol	13.099	4.12	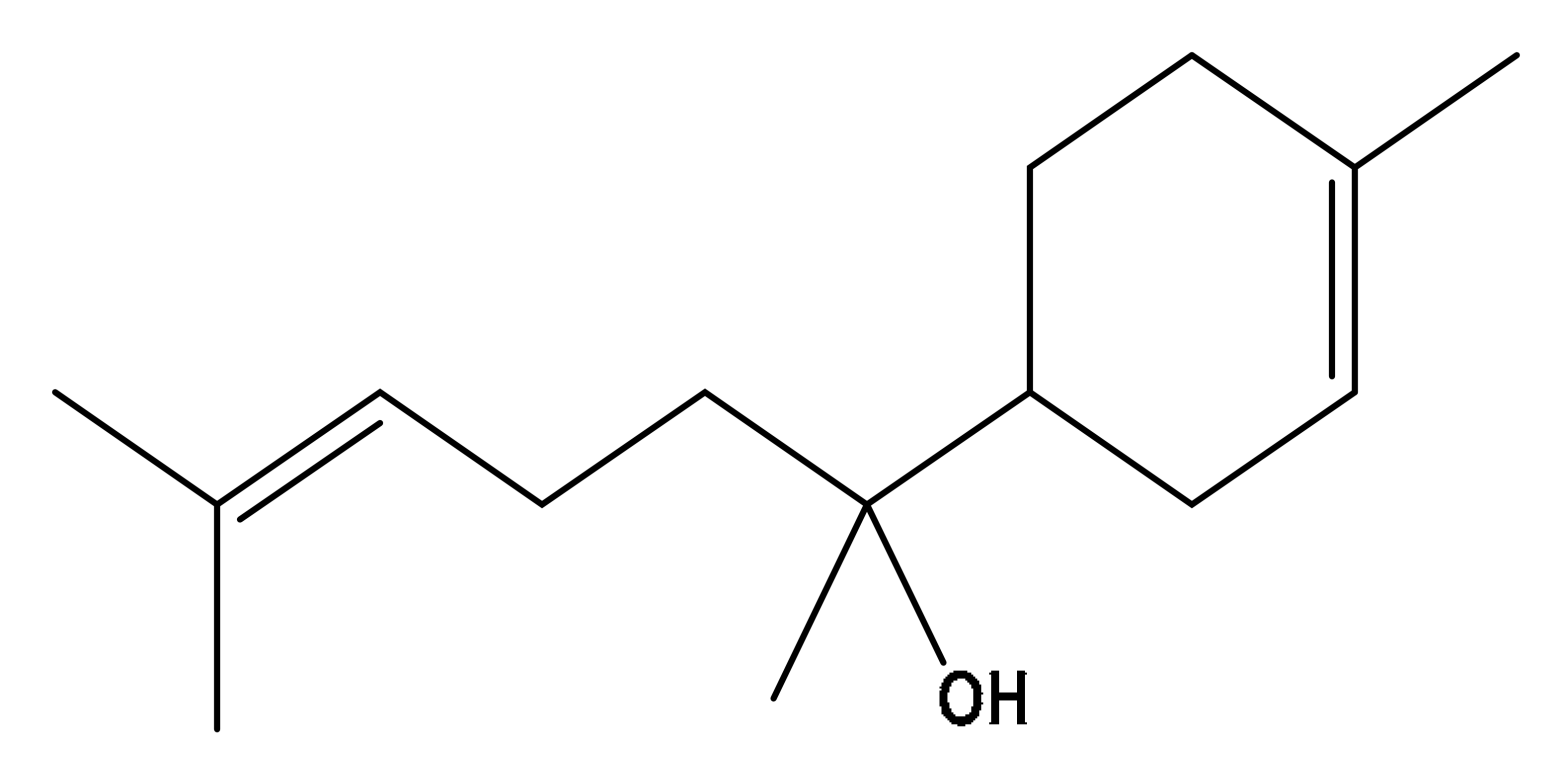	Oxygenated sesquiterpene	1682	1686	4
34	Juniper Camphor	13.174	0.64	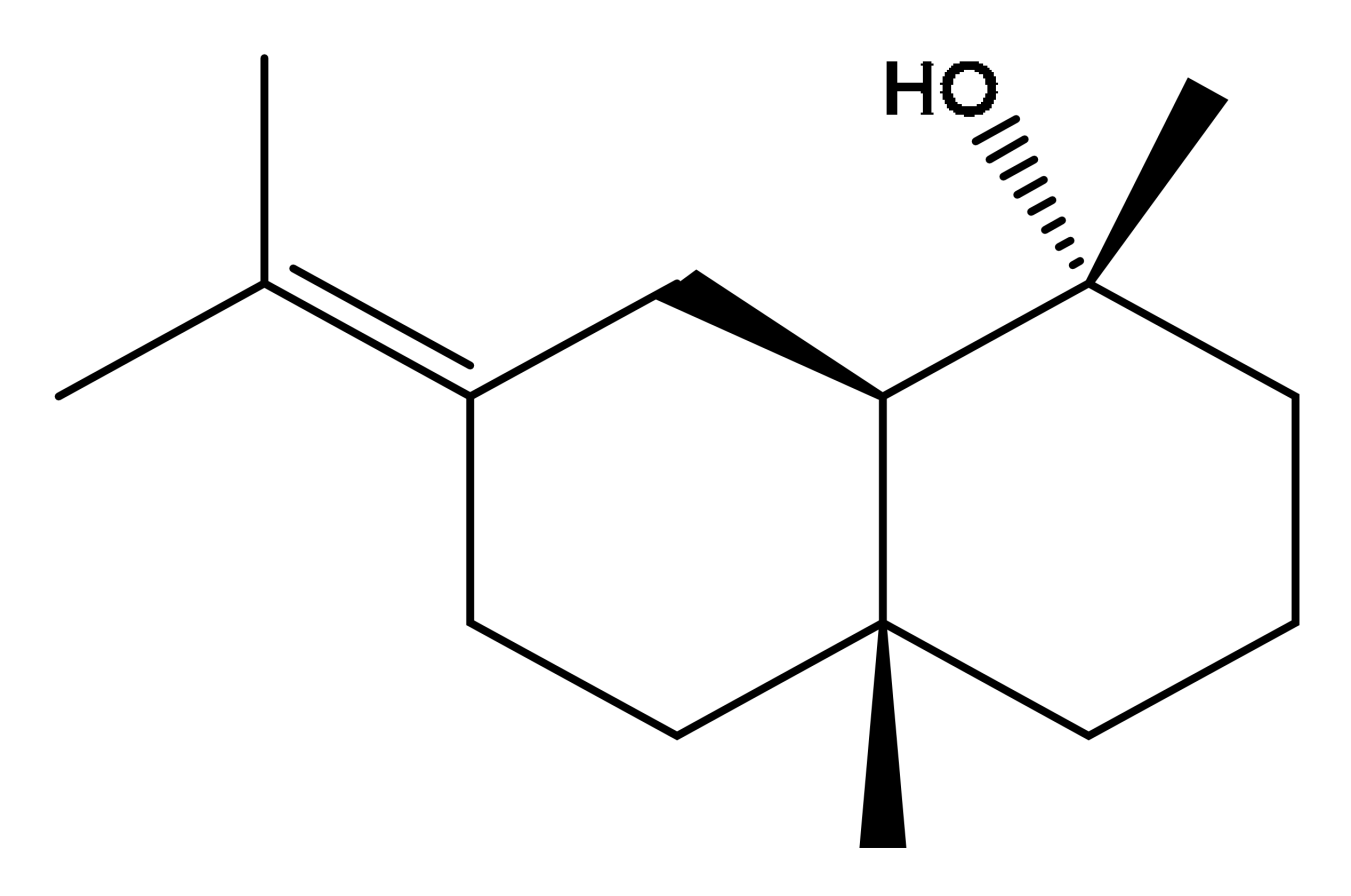	Oxygenated sesquiterpene	1665	1668	3
Sesquiterpene hydrocarbons				49.78%				
Oxygenated sesquiterpenes				41.14%				
Oxygenated monoterpenes				7.17%				
Monoterpene hydrocarbons				1.27%				
TOTAL				99.36%				

RI lit: literature retention indices from NIST and Adams databases for low-polarity columns [53,54]. RI exp: retention index determined on a RTX-5 capillary column using a homologous series of C8–C20.

### 3.2. Inhibition of Carbohydrate- and Lipid-Hydrolyzing Enzymes

**Figure 2:**
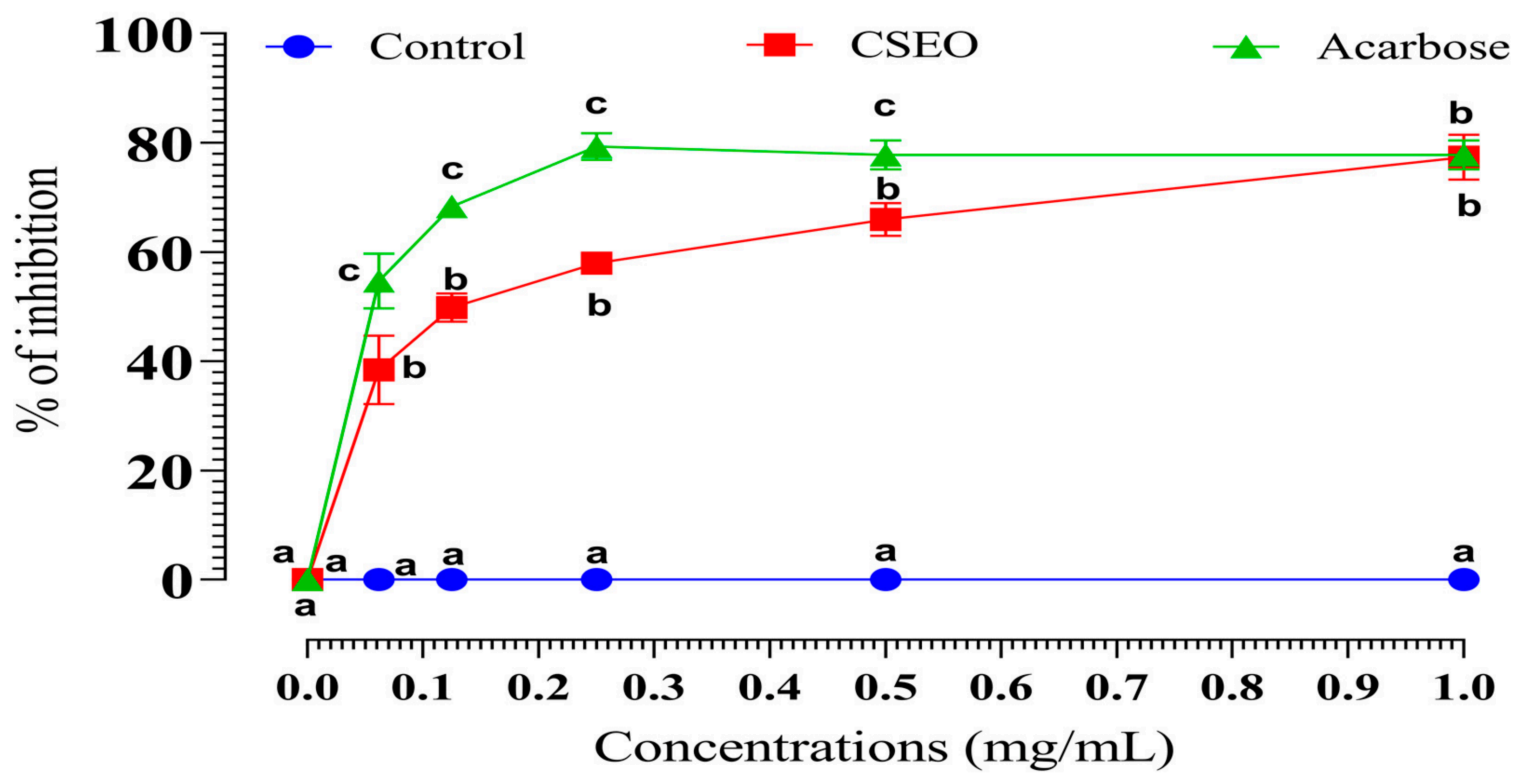
Comparative in vitro inhibition of α-amylase by CSEO and acarbose. Data represent mean ± SEM (*n* = 3). Graphs marked by different letters (a, b, c) are significantly different (the significance started at *p* < 0.05).

Functioning as the primary hydrolase catalyst for polysaccharide depolymerization within the gastrointestinal tract, α-amylase serves as a physiologically indispensable enzyme in carbohydrate assimilation [55]. Given its critical role in liberating glycemic substrates, targeted enzymatic inhibition of α-amylase represents a viable therapeutic strategy for mitigating postprandial hyperglycemia through delayed carbohydrate hydrolysis [55]. Figure 2 depicts the in vitro inhibitory effects on pancreatic α-amylase activity, with acarbose serving as the reference inhibitor.

Kinetic analysis revealed CSEO exhibits potent α-amylase inhibition (IC_50_ = 480 ± 5 μg/mL), confirming significant enzymatic inhibition. However, comparative assessment demonstrated reduced inhibitory potency relative to acarbose (IC_50_ = 382 ± 3 μg/mL), indicating a 1.26-fold efficacy gap (Table 2).

**Figure 3:**
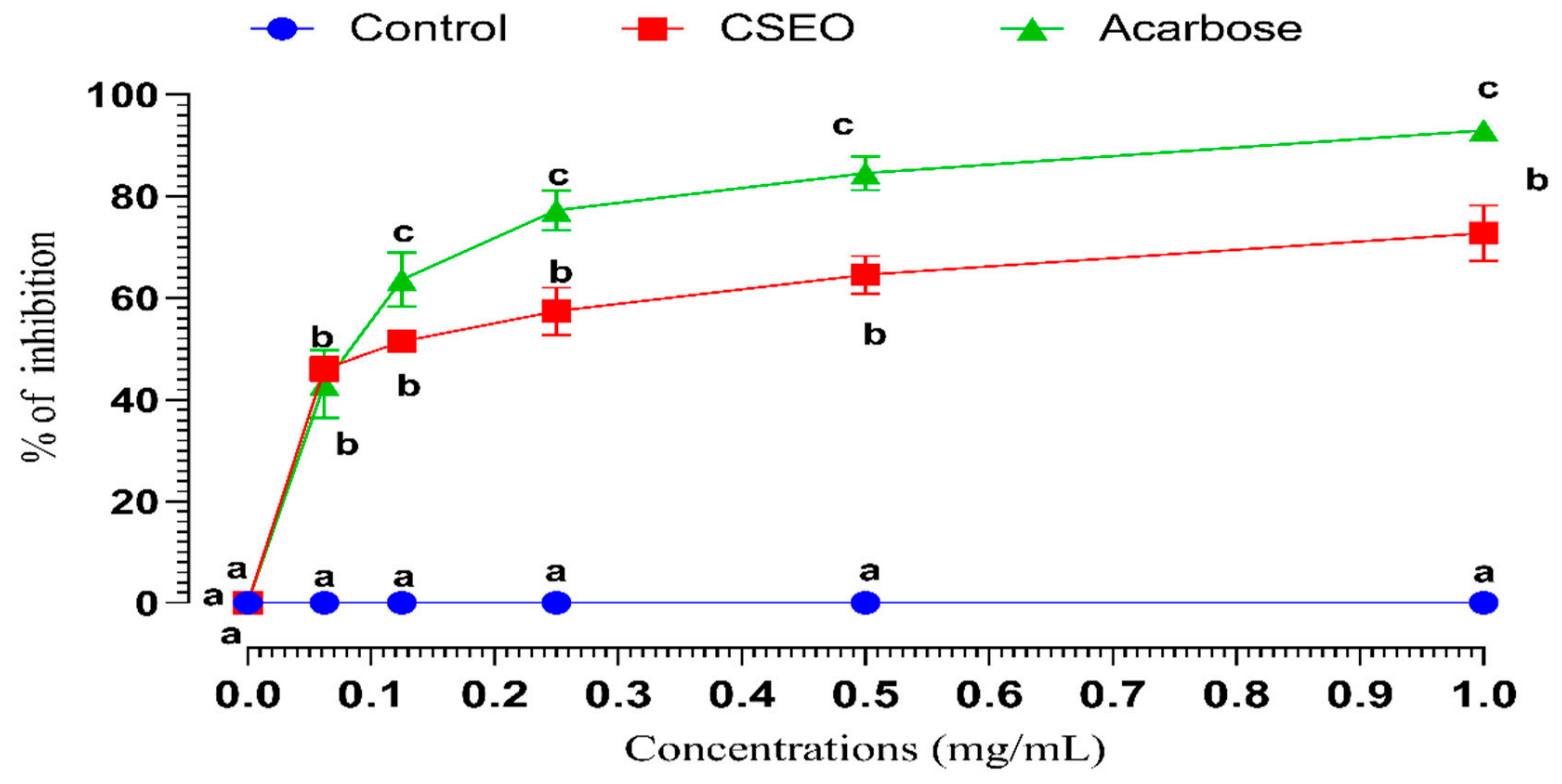
Comparative in vitro inhibition of α-glucosidase by CSEO and acarbose. Data represent mean ± SEM (*n* = 3). Graphs marked by different letters (a, b, c) are significantly different (the significance started at *p* < 0.05).

Localized within the brush border epithelium of the small intestinal mucosa, α-glucosidase catalyses the terminal hydrolysis of complex carbohydrates into absorbable monosaccharides. Pharmacological inhibition of this membrane bound enzyme represents a validated therapeutic strategy to attenuate postprandial hyperglycemia by limiting glucose absorption, thereby mitigating diabetes progression through reduced glycemic excursions [56]. Figure 3 illustrates the dose-responsive suppression of α-glucosidase activity of acarbose and CSEO, with maximal inhibition (>70%) attained at the highest test concentration (1.0 mg/mL).

**Figure 4:**
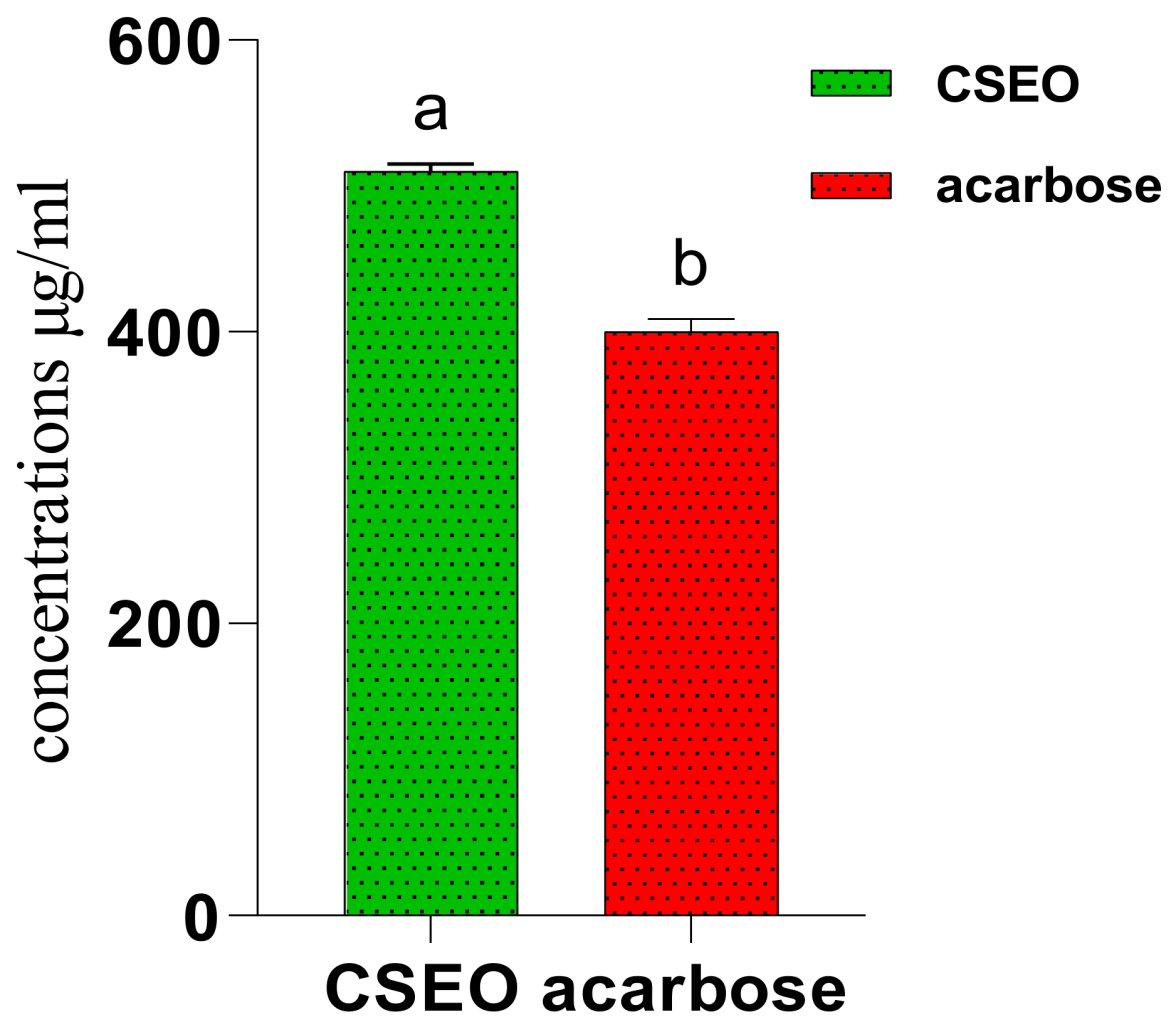
The IC_50_ values (μg/mL) for the inhibition of α-glucosidase by CSEO and acarbose. Data are presented as mean ± SEM (*n* = 3) Graphs marked by different letters (a, b) are significantly different (the significance started at *p* < 0.05).

Comparative analysis (Figure 4) revealed acarbose exhibited significantly stronger α-glucosidase inhibition (IC_50_ = 400 ± 9 μg/mL) than CSEO (IC_50_ = 510 ± 5 μg/mL).

**Figure 5:**
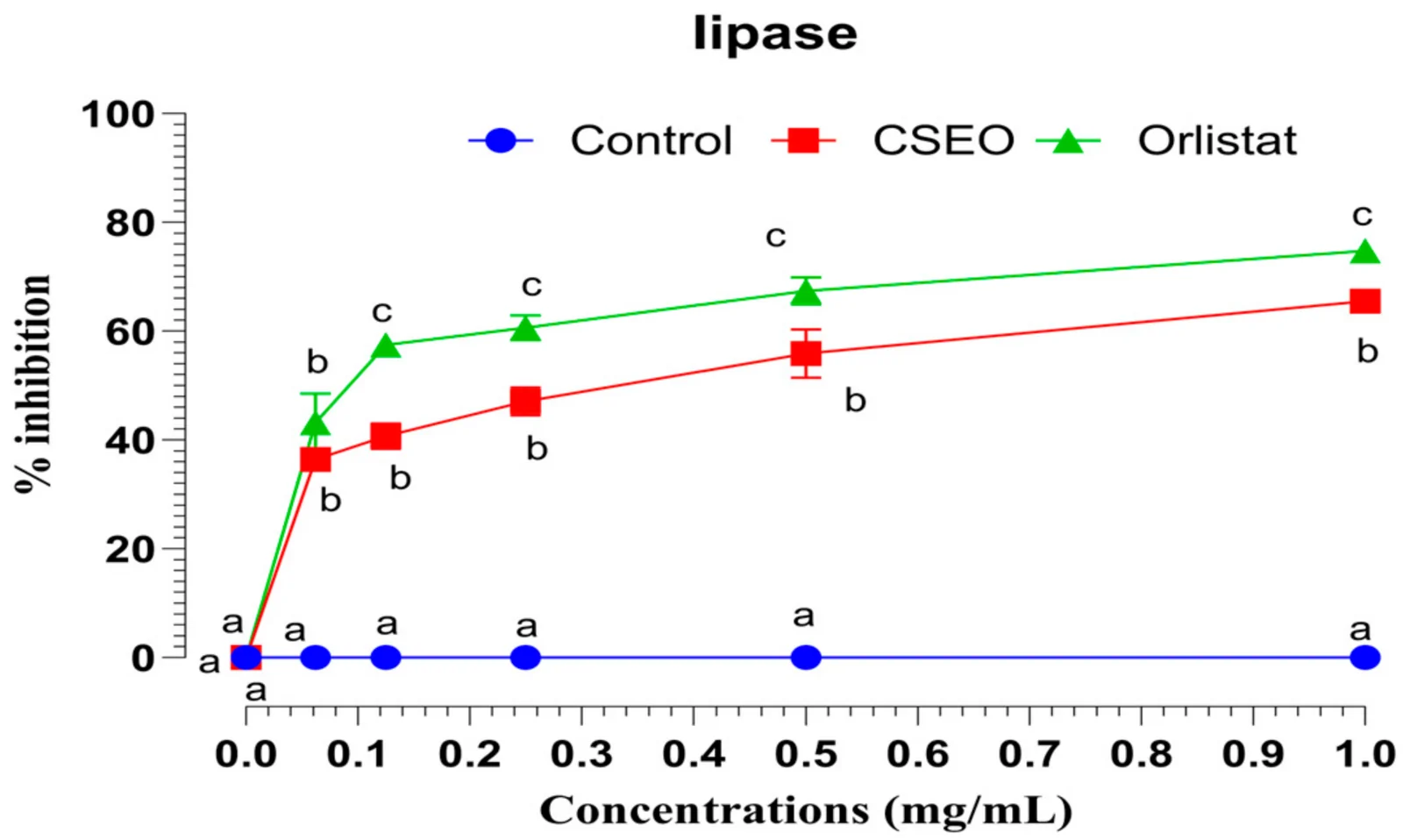
Comparative in vitro inhibition of Lipase by CSEO and Orlistat. Data represent mean ± SEM (*n* = 3). Graphs marked by different letters (a, b, c) are significantly different (the significance started at *p* < 0.05).

Pancreatic triacylglycerol lipase is a major enzyme involved in dietary lipid digestion in the small intestine, catalyzing the hydrolysis of triglycerides into absorbable free fatty acids and monoacylglycerols [38], In the present study, the inhibitory activity of *Cannabis sativa* L. essential oil (CSEO) against porcine pancreatic lipase was evaluated in vitro. As shown in Figure 5 and Table 3, CSEO exhibited a concentration-dependent inhibitory effect on pancreatic lipase, with an IC_50_ value of 589 ± 5 μg/mL. The reference inhibitor, orlistat, showed greater inhibitory activity, with an IC_50_ value of 490 ± 7 μg/mL, these results indicate that CSEO possesses in vitro pancreatic lipase inhibitory activity, although its activity was lower than that of the reference inhibitor.

Attenuation of enzymatic carbohydrate hydrolysis represents a clinically validated therapeutic strategy for mitigating postprandial hyperglycemia and managing dyslipidemia, primarily through delayed intestinal glucose absorption and reduced lipogenesis [57]. Research indicates that *Cannabis sativa* L. extracts exhibit antidiabetic properties, evidenced by enzyme inhibition in vitro activity [35,52,58,59], Our results corroborate earlier reports, revealing that *Cannabis sativa* L. essential oil act as inhibitors of α-amylase and α-glucosidase enzymes. The interpretation of these findings nonetheless warrants a degree of caution regarding the attribution of enzyme-inhibitory activity to specific chemical constituents. Given that CSEO was evaluated as an intact volatile matrix rather than as isolated fractions, the observed inhibitory effects reflect the collective action of the essential oil rather than the confirmed contribution of any single terpene. Attribution of activity to particular compounds, such as β-caryophyllene or other major constituent, is therefore inferential, resting on their relative abundance within the GC-MS profile and on convergence with independent reports describing inhibitory activity for these compounds in isolation [60]. Such correlative reasoning is widely employed in essential oil pharmacology, yet it does not, by itself, establish causality, since essential oils constitute complex multi-component mixtures in which major and minor constituents may interact additively, synergistically, or antagonistically to shape the net biological outcome [61]. Establishing a more definitive structure activity relationship for CSEO would therefore require complementary approaches, including bioassay-guided fractionation, evaluation of purified major constituents, or enzyme kinetic analyses.

Obesity, driven by chronic energy intake exceeding expenditure, causes pathological adipose tissue accumulation, hyperfunction, and lipid metabolic dysregulation. A method for managing this condition is the attenuation of luminal lipid hydrolysis, achieved through inhibition of pancreatic lipase [62,63]. As this enzyme mediates the digestion of ingested fats, its suppression is critical [64,65]. Orlistat, a pharmacological lipase inhibitor employed for obesity treatment, may induce significant adverse events, notably renal pathologies [66]. The present investigation explored the effect of elicitation on the pancreatic lipase-inhibiting properties of *Cannabis sativa* L. essential oils. Compared to Zengin et al.’s findings, the essential oils in our study exhibited reduced efficacy in lipase inhibition [52]. Notably, β-caryophyllene, identified as the major constituent of CSEO in the present analysis, has independently been reported to display potent pancreatic lipase inhibitory activity [67] a finding consistent with its dominant proportion in our oil. This concordance, while suggestive of a contributory role for β-caryophyllene, should be interpreted with the same caution outlined above, as the comparatively lower lipase-inhibitory efficacy observed here relative to Zengin et al. [52] also illustrates how variation in the relative proportions of minor, co-occurring constituents can modulate whole oil bioactivity independently of the dominant compound.

Overall, the present findings demonstrate that CSEO possesses in vitro inhibitory activity against α-amylase, α-glucosidase, and pancreatic lipase. The results, together with the chemical characterization of the oil, provide preliminary evidence for the biological activity of this particular essential-oil preparation. However, the findings do not establish that any individual terpene is responsible for the observed effects, nor do they demonstrate clinical efficacy. Further investigations involving isolated constituents, defined fractions, reconstituted mixtures, and appropriate in vivo models will be necessary to determine the contribution of individual compounds and to clarify the mechanisms underlying the observed activities.

### 3.3. Hemoglobin Glycation Inhibition

**Figure 6:**
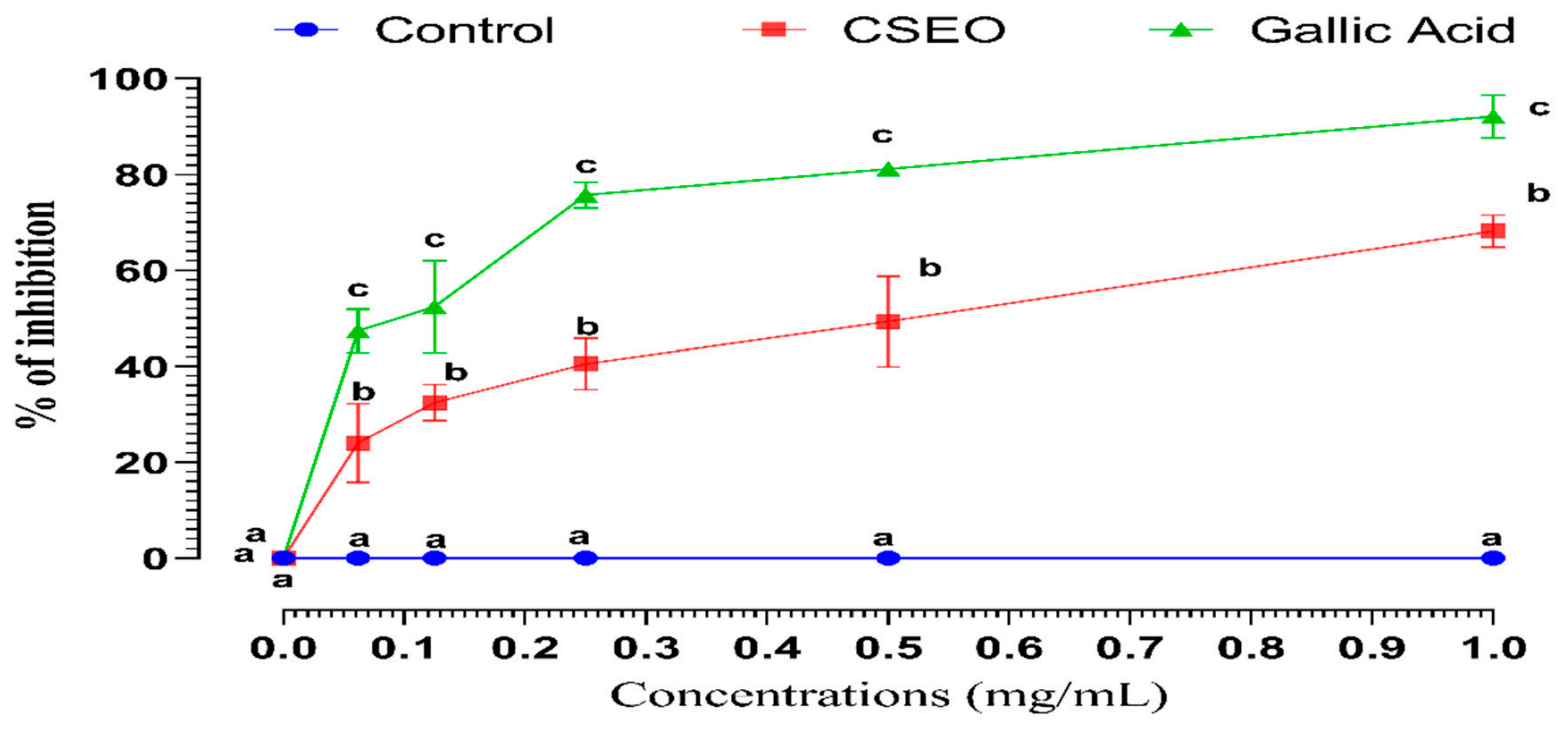
Inhibitory effect of CSEO and Gallic acid (positive control) on glycation activity of hemoglobin. Results are shown as mean ± SEM (*n* = 3). Graphs marked by different letters (a, b, c) are significantly different (the significance started at *p* < 0.05).

As demonstrated in Figure 6 and Table 4, CSEO inhibited hemoglobin glycation in a concentration-dependent manner, with glycosylation levels declining proportionally to treatment concentration. Quantitative analysis revealed moderate antiglycation activity for CSEO (IC_50_ = 610 ± 5 μg/mL), while gallic acid exhibited greater efficacy (IC_50_ = 412 ± 3 μg/mL).

### 3.4. Effect of CSEO on Multiple Stages of Albumin Glycation

In biological systems, monosaccharides such as glucose and fructose can participate in non-enzymatic reactions with proteins, leading to modifications in protein structure and function. This process, known as glycation, drives the formation and accumulation of advanced glycation end products (AGEs). Attachment of AGEs to receptors such as RAGE promotes the breakdown of functional proteins and tissue damage [38,68] and progressive AGE accumulation is recognized as a major factor in the onset and progression of diabetes-related complications. Glycation has also been implicated in accelerated aging, a phenomenon termed “glycation stress” that has attracted growing scientific interest [37]. Molecules combining antiglycation and antioxidant activity have shown superior efficacy against diabetic pathogenesis relative to single-target agents [69]. While CSEO’s antioxidant properties have been previously established [43,51,70], the present work provides the first evidence that this oil can interfere with albumin glycation across multiple stages of the reaction cascade.

The data outlined in Table 5 reveal that CSEO exert a pronounced inhibitory effect on albumin glycation throughout multiple phases of the glycation process. During the early phase, assessed via the fructosamine content (TBA assay), CSEO showed inhibition with IC_50_ values of 480 ± 2 μg/mL, which is comparable to the standard control aminoguanidine (AG) with IC_50_ values of 452 ± 6 μg/mL. Suppression was also evident in the intermediate stage, measured by protein carbonyl compound generation (DNPH assay), yielding IC_50_ values of 456 ± 4 μg/mL for CSEO versus 463 ± 3 μg/mL for AG. Furthermore, analysis of advanced glycation end-products (AGEs) by fluorescence in later stages confirmed CSEO’s inhibitory capacity, showing IC_50_ values of 460 ± 4 μg/mL (CSEO) and 450 ± 5 μg/mL (AG). Importantly, glycation is a critical mechanism that promotes alterations in protein conformation by increasing amyloid cross β-structures, which facilitate protein aggregation a hallmark of amyloidosis implicated in multiple chronic disorders such as diabetes [71]. To explore this aspect, our study evaluated the ability of CSEO to prevent the aggregation of glycated albumin using Congo red as an amyloid marker. CSEO demonstrated a strong inhibitory effect on amyloid formation, with an IC_50_ value of 430 ± 6 μg/mL compared to 460 ± 4 μg/mL for aminoguanidine (Table 5).

The accumulation of advanced glycation end products (AGEs) activates multiple receptor-mediated signaling pathways, ultimately disrupting essential biological processes and cellular function to induce cell death [72]. In the present study, the tested essential oil demonstrated noteworthy efficacy in inhibiting AGE formation in a cell-free BSA-glucose model. These observations are consistent with previous reports showing that terpenes can inhibit AGE generation [73,74]. Separately, β-caryophyllene, a prominent terpenoid component of the oil, has been reported to attenuate RAGE activation in hepatic models [75], suggesting that certain essential oil constituents may also have the potential to modulate AGE–RAGE signaling in a cellular context; this remains to be confirmed for CSEO itself. It should be noted, however, that inhibition of AGE formation in this type of assay may arise from more than one mechanism. In addition to a specific interaction with glycation intermediates, the antiglycation activity observed here could partly reflect the intrinsic physicochemical properties of the essential oil, including its antioxidant/radical-scavenging capacity and the potential of reactive carbonyl or terpenoid constituents to directly scavenge free sugars or reactive dicarbonyl intermediates, rather than a targeted biological antiglycation mechanism [76,77]. Indeed, several studies have reported no correlation between antioxidant capacity and antiglycation activity, suggesting that plant-derived compounds may act through independent pathways rather than a single, shared mechanism [68,78]. Since Maillard chemistry proceeds through multiple stages (early Schiff base/Amadori rearrangement, oxidative intermediate formation, and late-stage AGE cross-linking) each of which is differentially sensitive to the surrounding chemical environment [79,80,81]. Complementary assays (dicarbonyl-trapping assay) [82]), oxidation-independent glycation models [82] or antioxidant-activity correlation analysis [82] would be needed to clarify the specific stage(s) targeted.

Collectively, these findings indicate that CSEO possesses antiglycation activity in vitro, warranting further mechanistic investigation into whether this reflects biologically specific inhibition, non-specific physicochemical interference with the Maillard reaction, or a combination of both, before conclusions can be drawn regarding its therapeutic relevance in glycation-related conditions such as diabetes.

### 3.5. Docking

**Figure 7:**
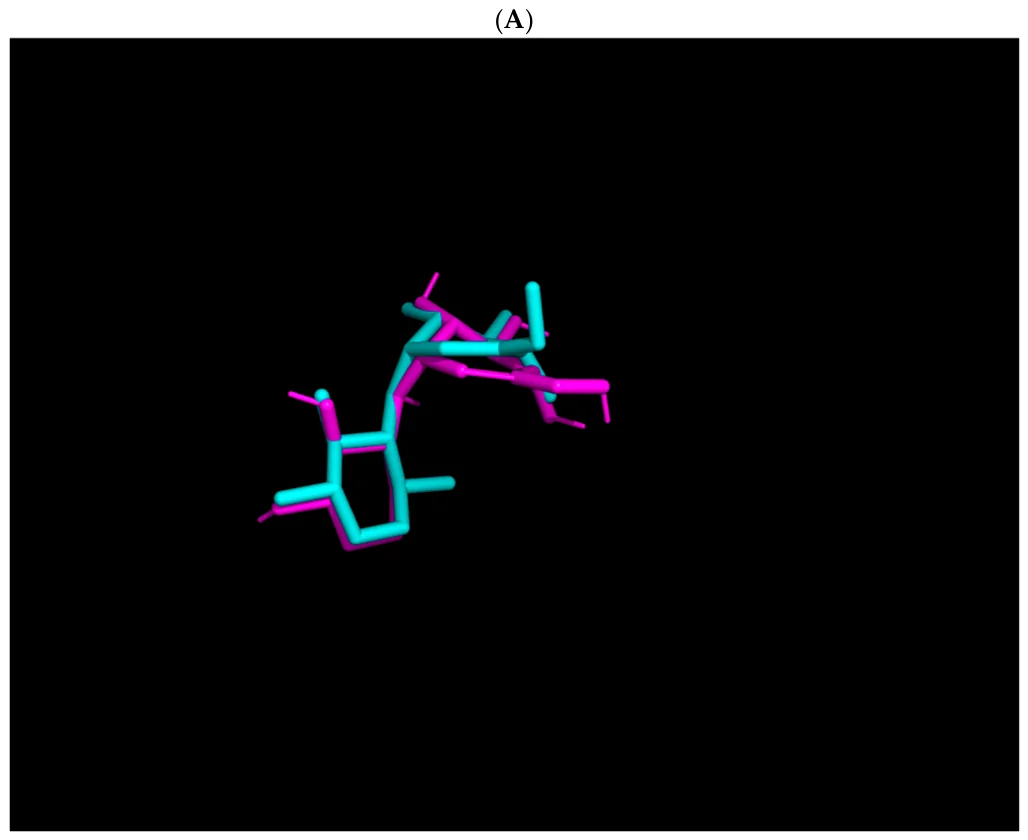

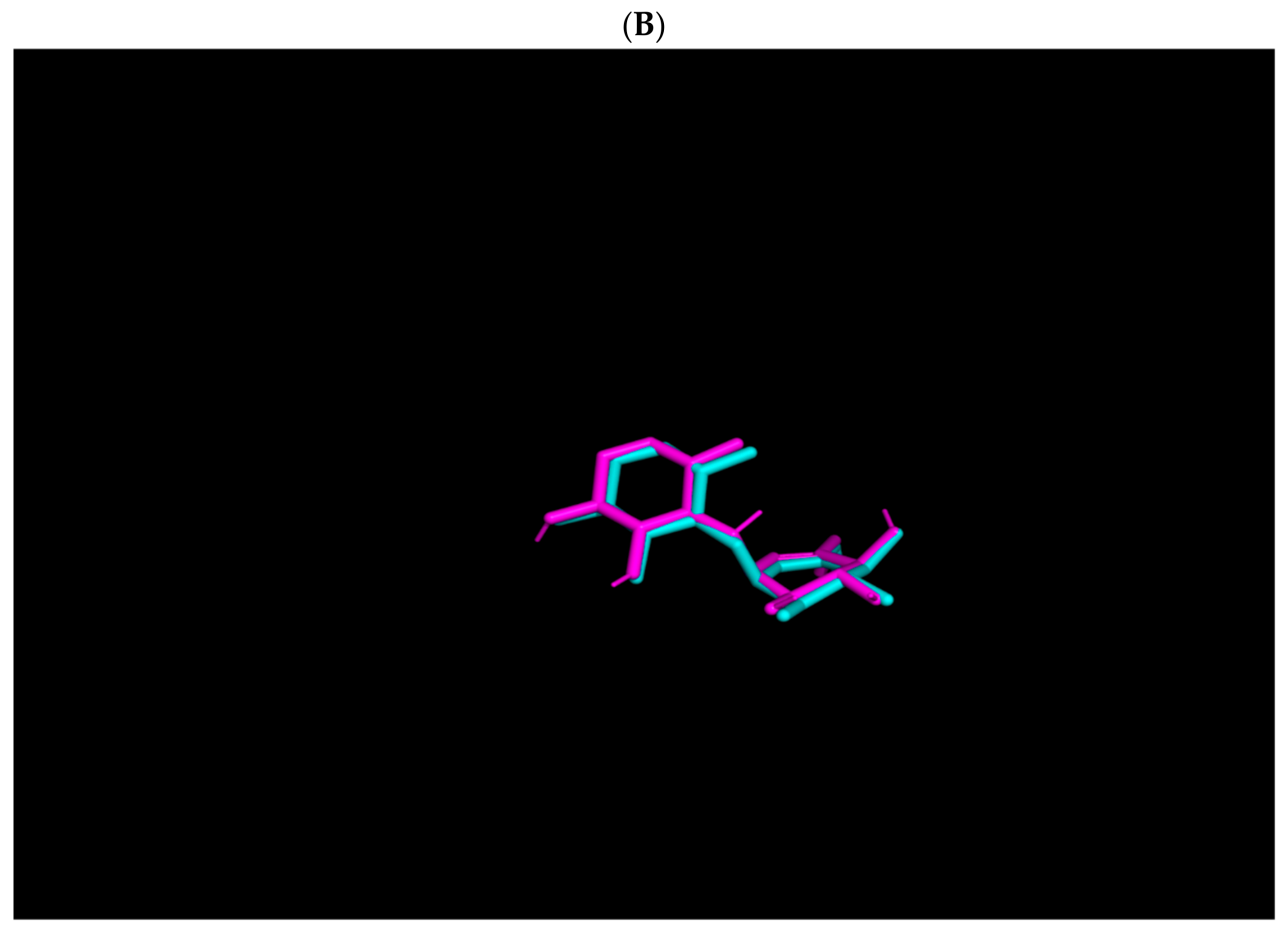
Superposition of native (cyan) and redocked (magenta) acarbose in alpha-amylase (**A**) and alpha-glucosidase (**B**).

The docking protocol was validated by redocking the crystallographic of acarbose into the active sites of α-amylase and α-glucosidase. The recalculated redocking procedure yielded RMSD values of 0.771 Å for α-amylase (PDB ID: 1B2Y) and 0.304 Å for α-glucosidase (PDB ID: 5NN8). Both values were substantially below the commonly accepted threshold of 2.0 Å, indicating that the protocol accurately reproduced the experimentally determined ligand poses. The corresponding superposition analysis (Figure 7A,B) further confirmed the close agreement between the crystallographic and redocked conformations, thereby demonstrating the reliability of the docking protocol for predicting ligand-binding modes in both enzymatic systems.

The inhibition of carbohydrate-digesting enzymes, such as Alpha-Amylase and Alpha-Glucosidase, represents a key therapeutic strategy for managing postprandial hyperglycemia in patients with type 2 diabetes. The molecular docking results revealed strong binding affinities for several natural compounds, indicating their potential as antidiabetic agents (Table 6).

For Alpha-Amylase, the compounds exhibiting the strongest predicted binding affinities, based on the Glide gscore, are Bulnesol (−4.809 kcal/mol), followed by 2-Pinen-4-one (−4.762 kcal/mol), and Isoaromadendrene Epoxide (−4.756 kcal/mol). These high negative values suggest a stable and favorable interaction with the enzyme’s active site.

Regarding Alpha-Glucosidase, the top binders are Camphol (−5.435 kcal/mol), Trans-Pinocarveol (−5.264 kcal/mol), and 2-Fenchanol (−5.008 kcal/mol). It is notable that the gscores for Alpha-Glucosidase are generally more negative than those observed for Alpha-Amylase, potentially indicating a better overall affinity of the tested compounds for this enzyme.

**Figure 8:**
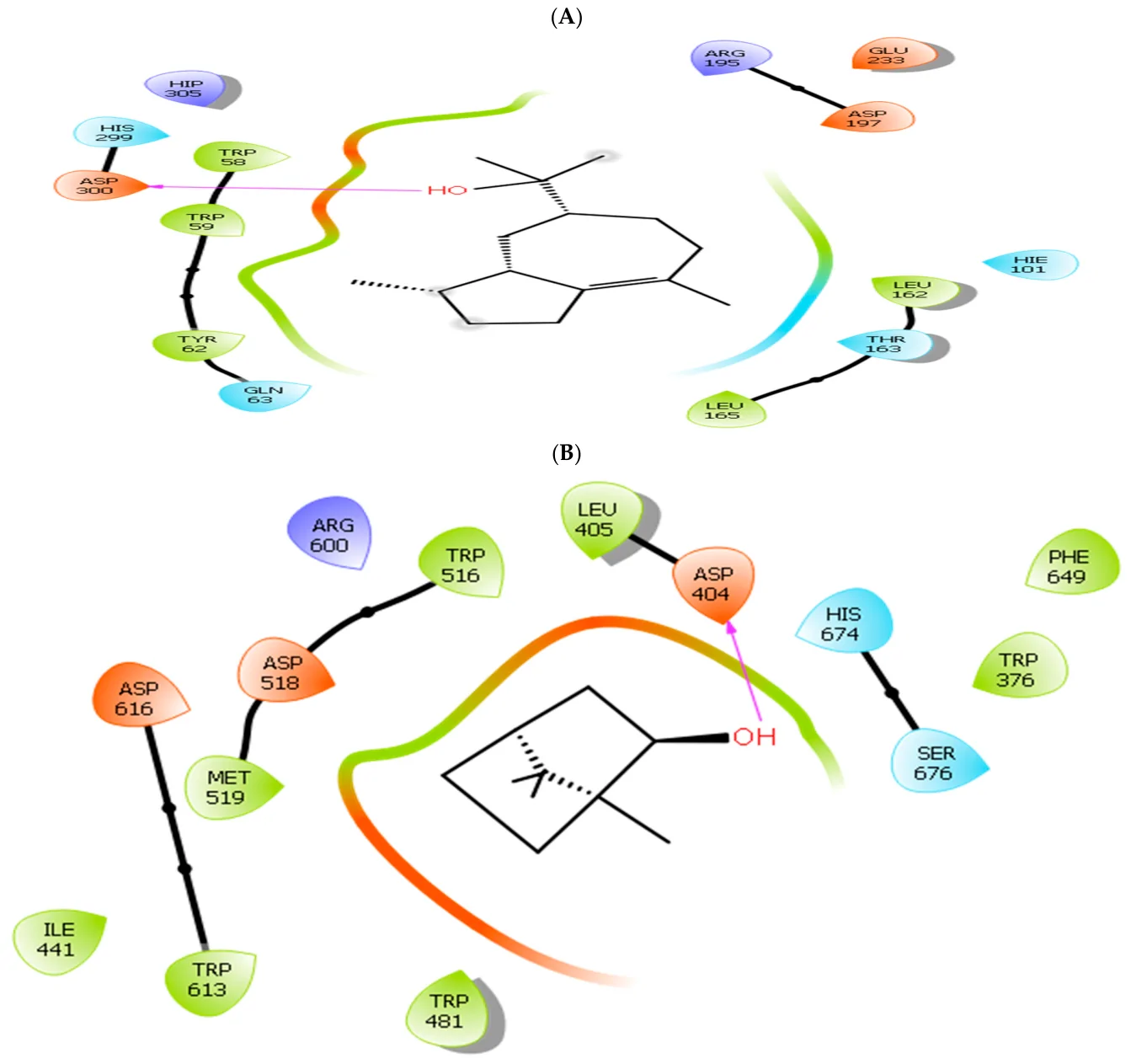
The 2D viewer of ligand interactions with the active site. (**A**): Bulnesol interactions with the active site of alpha-amylase, (**B**): camphol interactions with active site of alpha-glucosidase.

**Figure 9:**
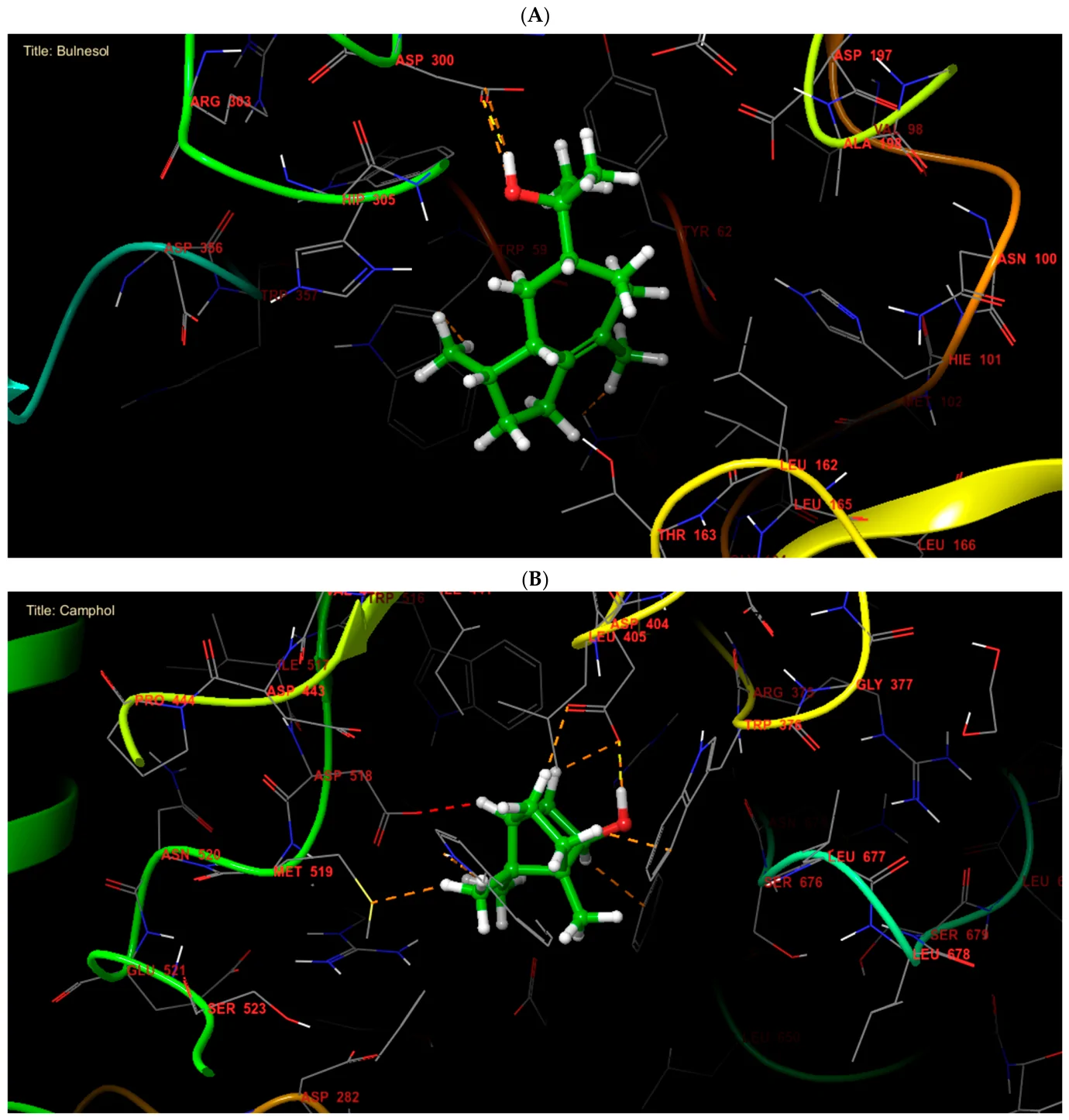
The 3D viewer of ligand interactions with the active site. (**A**): Bulnesol interactions with the active site of alpha-amylase, (**B**): camphol interactions with active site of alpha-glucosidase.

The 2D and 3D interaction between the lines and the active sites showed that Bulnesol formed a single hydrogen bond with the ASP 300 residue in the alpha-amylase active site (Figure 8A and Figure 9A). Similarly, camphol also formed a single hydrogen bond with the ASP 404 residue in the alpha-glucosidase active site (Figure 8B and Figure 9B).

The pharmacokinetic and physicochemical properties of the identified compounds were evaluated using the QikProp module. Since this module is designed to predict molecular physicochemical and ADME properties from individual three-dimensional structures, the analysis was performed at the level of individual constituents rather than on the essential oil mixture [83,84]. Most compounds complied with Lipinski’s rule of five, and the majority showed no violations of this rule. However, this criterion should be interpreted only as a filter for therapeutic potential and not as proof of safety or confirmed oral bioavailability Table 7.

The predicted human oral absorption parameter was 100% for all molecules. This result should be considered an estimate derived from the model, subject to its assumptions, and should not be interpreted as measured absorption, systemic exposure, absolute bioavailability, or clinical tolerability. Oral bioavailability depends not only on intestinal absorption, but also on dissolution, formulation, intestinal and hepatic metabolism, transport processes, dose, and the composition of the administered preparation. Thus, current results indicate a favorable predicted absorption profile for the isolated molecules, but they do not allow for the determination of their actual bioavailability.

To provide a preliminary safety assessment, the QPlogHERG descriptor was also examined. QPlogHERG is a logarithmic estimate, predicted by QikProp, of the IC_50_ for the blockade of hERG potassium channels, an in vitro risk associated with delayed cardiac repolarization and potential QT interval prolongation [83,85,86]. For the molecules identified in *Cannabis sativa* L. Essential Oil, the QPlogHERG values ranged from −4.727 to −1.645, with a mean of −2.803 and a median of −2.601. The least negative values were observed for isoaromadendrene epoxide (range: −2.756 to −1.645; mean: −2.361) and α-pinene epoxide (range: −2.171 to −1.707; mean: −1.940), while α-farnesene (−4.727), β-farnesene (−4.704) and α-bisabolol (−4.151) showed more negative predicted values. Since QPlogHERG is a computer-generated estimate and no data regarding hERG IC_50_, cytotoxicity, genotoxicity, hepatotoxicity, reproductive toxicity, or in vivo toxicity were experimentally determined in this study, these values should be considered preliminary risk indicators rather than proof of a compound’s safety or toxicity.

It is important to note that the predictions were generated for isolated molecules and cannot be directly extrapolated to the essential oil mixture. The biological activity and toxicity of an essential oil can depend on the concentrations of its constituents, their additive, synergistic, or antagonistic interactions, their chemical stability, and the route and dose of exposure [87]. Therefore, the computer modeling results can be used to prioritize constituents for further testing but do not constitute a comprehensive safety assessment of the essential oil. Experimental confirmation should include, where ap-propriate, cytotoxicity testing on relevant human cell models, an in vitro hERG assay or a validated cardiac ion channel assay, and evaluation of the whole essential oil at exposure levels relevant to the intended use. The composition of the essential oil should also be quantified prior to these tests, as variations in the proportions of constituents can alter its efficacy and toxicity.

The molecular docking results demonstrate that several tested natural compounds, primarily terpenes, possess a strong affinity for the active sites of Alpha-Amylase and Alpha-Glucosidase. These enzymes are crucial pharmacological targets for the treatment of type 2 diabetes, as their inhibition slows the digestion of complex carbohydrates, thereby reducing glucose absorption and postprandial hyperglycemia [88,89].

The particularly high affinity observed for Alpha-Glucosidase, with Camphol achieving a gscore of −5.435 kcal/mol, is consistent with the literature highlighting the antidiabetic potential of monoterpenes and sesquiterpenes. In silico and in vitro studies have shown that compounds structurally related to Camphol and 2-Fenchanol, such as camphor and fenchone, exert significant inhibition of Alpha-Glucosidase and Alpha-Amylase [90]. These terpenes, often found in essential oils, are recognized for their ability to interact with key amino acid residues in the enzyme active sites, primarily through hydrophobic interactions [90].

Bulnesol, the most active compound against Alpha-Amylase (−4.809 kcal/mol), is a sesquiterpene whose biological potential is widely studied. Although direct data on its enzymatic inhibition may vary, its classification as a sesquiterpene, a class known for its antidiabetic and antioxidant activities, supports the relevance of this docking result [91,92].

The observation that gscores are generally more favorable for Alpha-Glucosidase than for Alpha-Amylase (Camphol is the best binder for Alpha-Glucosidase, while Bulnesol is the best for Alpha-Amylase, with a less negative score) could indicate a structural selectivity of the ligands for the Alpha-Glucosidase binding pocket. This selectivity is an important factor in drug development, as selective inhibition of intestinal Alpha-Glucosidase is often preferred over pancreatic Alpha-Amylase inhibition to minimize gastrointestinal side effects.

In conclusion, the molecular docking results suggest that compounds such as Camphol, Trans-Pinocarveol, and Bulnesol are promising candidates for in vitro and in vivo evaluation as inhibitors of key carbohydrate metabolism enzymes.

The evaluation of ADME (Absorption, Distribution, Metabolism, Excretion) properties is a fundamental step in drug discovery, allowing for the filtering of compounds likely to fail during clinical phases due to pharmacokinetic issues [93]. The in silico analysis performed with QikProp characterized the oral bioavailability profile of the studied compounds.

Lipinski’s Rule of Five (Ro5) is a drug-likeness filter that predicts the likelihood of good oral absorption. A Rule Of Five score of 0, as is the case for the majority of molecules in Table 6, indicates full compliance with the four Lipinski criteria (mol MW < 500 Da, QPlogPo/w < 5, donorHB ≤ 5, accptHB ≤ 10). The molecular weight (mol MW) of all compounds is well below 500 Da, and the counts of hydrogen bond donors and acceptors are low, which is typical for terpenes and favorable for membrane permeability [94].

The octanol/water partition coefficient (QPlogPo/w) is a key indicator of lipophilicity. The predicted values are mostly within the optimal range of −2.0 to 6.5 for good oral absorption. The most lipophilic compounds, such as Valencene and alpha-Bulnesene (QPlogPo/w ≈ 5.452), are sesquiterpene hydrocarbons that maintain high lipophilicity but remain within the acceptable limits of the Ro5.

Aqueous solubility (QPlogS) is a critical factor for absorption. The QPlogS values are all negative (ranging from −2.053 to −6.561), which is expected for lipophilic compounds like terpenes. However, the least negative values, such as those for 2-Fenchanol (−2.053) and Trans-Pinocarveol (−2.12), suggest relatively better aqueous solubility, which is beneficial for dissolution and gastrointestinal absorption [95].

Caco-2 cell permeability (QPPCaco) is an in-silico model for intestinal permeability. High values (above 500 nm/s) are generally considered excellent. The majority of compounds, particularly sesquiterpene hydrocarbons like Valencene and D-Limonene (QPPCaco ≈ 9906 nm/s), exhibit very high predicted permeability, suggesting near-complete intestinal absorption [96].

The brain/blood partition coefficient (QPlogBB) predicts a compound’s ability to cross the blood-brain barrier (BBB). The recommended range for good CNS penetration is −3.0 to 1.2. All compounds show QPlogBB values within this range (between 0.041 and 1.012), indicating a potential for CNS penetration. The highest values, close to 1.0 (Valencene and alpha-Bulnesene), suggest easier penetration, which could be relevant if a central action is desired [97].The predicted Percent Human Oral Absorption is 100% for all compounds, reinforcing the idea that these molecules are excellent candidates for oral administration. The overall ADME profile, characterized by low molecular weight, moderate lipophilicity, and high permeability, positions these natural compounds as promising drug-like entities for further preclinical studies.

## 4. Conclusions

The present study demonstrates that *Cannabis sativa* L. essential oil (CSEO), characterized by a sesquiterpene-rich chemical profile, exhibits measurable in vitro digestive-enzyme inhibitory and antiglycation activities. CSEO inhibited α-amylase, α-glucosidase, and pancreatic lipase, while also showing inhibitory effects in hemoglobin and albumin glycation models, indicating activity against different stages and aspects of protein glycation. Among the digestive enzymes investigated, the observed effects varied according to the target enzyme, highlighting the importance of considering the specific biological system when evaluating the activity of CSEO.

Molecular docking analysis identified several CSEO constituents with favorable predicted interactions with α-amylase and α-glucosidase, providing possible molecular-level explanations for part of the observed enzyme-inhibitory activity. However, these computational predictions do not constitute direct evidence of enzyme binding or inhibition, and the biological effects observed for the whole essential oil cannot be attributed to individual constituents solely on the basis of their abundance or docking scores.

Overall, the findings provide preliminary in vitro evidence supporting the biological activity of CSEO in digestive-enzyme inhibition and antiglycation models. Nevertheless, the present results should not be interpreted as evidence of therapeutic efficacy. Further studies involving purified constituents, defined fractions or reconstituted mixtures, enzyme-kinetic and mechanistic investigations, as well as appropriate in vivo models, are required to clarify the active components, underlying mechanisms, safety, and biological relevance of the observed effects.

## Figures and Tables

**Table 2:** IC_50_ value (μg/mL) of inhibition of α-amylase by CSEO and acarbose. Data are presented as mean ± SEM (*n* = 3).

	IC_50_ (μg/mL)
Acarbose	382 ± 3
CSEO	480 ± 5

**Table 3:** IC_50_ value (μg/mL) of inhibition of lipase by CSEO and Orlistat. Data are presented as mean ± SEM (*n* = 3).

	IC_50_ (μg/mL)
Orlistat	490 ± 7
CSEO	589 ± 5

**Table 4:** IC_50_ of hemoglobin antiglycation activity of CSEO and gallic acid. Data are presented as mean ± SEM (*n* = 3).

	IC_50_ (μg/mL)
Gallic acid	412 ± 3
CSEO	610 ± 5

**Table 5:** IC_50_ values of the multiple stages of albumin glycation. Data are presented as mean ± SEM (*n* = 3).

	IC_50_ (μg/mL)			
	Glycation Inhibition Reaction Assay			
	TBA	DNPH	Congo Red	AGEs
Aminoguanidine	452 ± 6	463 ± 3	460 ± 4	450 ± 5
CSEO	480 ± 2	456 ± 4	430 ± 6	460 ± 4

**Table 6:** Docking results of ligands in different receptors.

	Alpha-Amylase (PDB: 1B2Y)			Alpha-Glucosidase (PDB: 5NN8)		
Molecule	Glide Gscore	Glide Emodel	Glide Energy	Glide Gscore	Glide Emodel	Glide Energy
1,8-Cineole	−3.705	−26.51	−21.328	−4.687	−26.962	−21.164
2-Fenchanol	−4.332	−30.405	−23.277	−5.008	−28.59	−21.614
2-Pinen-4-one	−4.762	−27.71	−20.811	−4.634	−26.018	−20.769
alpha-Bergamotene	−3.011	−24.507	−20.694	−3.401	−25.274	−21.864
alpha-Bisabolol	−3	−29.57	−24.394	−3.088	−26.656	−21.862
alpha-Bulnesene	−3.887	−24.986	−20.282	−3.439	−24.068	−20.168
alpha-Copaen-11-ol	−4.658	−31.22	−23.516	−3.492	−23.794	−19.87
alpha-Farnesene	0.199	−20.941	−21.689	0.013	−20.961	−22.619
alpha-Gurjunene	−3.731	−25.057	−20.269	−3.363	−17.63	−14.501
alpha-Pinene epoxide	−3.843	−26.01	−20.781	−4.683	−27.721	−21.75
alpha-Terpineol	−4.211	−26.145	−20.812	−4.166	−31.073	−23.952
beta-Cymene	−4.239	−23.856	−18.843	−4.124	−25.229	−20.044
beta-Eudesmene	−3.858	−24.536	−19.473	−3.213	−20.585	−17.048
beta-Farnesene	1.588	−21.2	−24.55	0.382	−20.291	−22.501
beta-Linalool	−2.174	−22.696	−21.008	−2.535	−25.692	−23.34
Bulnesol	−4.809	−30.428	−23.395	−4.759	−32.833	−27.067
Camphol	−4.497	−32.351	−24.464	−5.435	−27.833	−21.781
Camphor	−4.376	−28.464	−21.878	−4.52	−21.071	−17.034
Caryophyllene	−3.526	−24.905	−20.293	−3.492	−20.912	−17.677
Cis-alpha-Bisabolene	−3.487	−25.246	−21.727	−3.419	−23.743	−19.752
Cis-Verbenol	−4.275	−25.867	−19.897	−4.684	−26.256	−19.813
Cubenol	−3.773	−29.385	−23.346	−4.77	−28.644	−22.364
D-Limonene	−3.72	−19.138	−15.793	−3.283	−20.025	−16.462
Epiglobulol	−3.896	−27.801	−22.298	−3.783	−24.546	−20.124
gamma-Eudesmol	−4.456	−29.39	−23.316	−3.407	−22.626	−19.206
gamma-Gurjunene	−3.566	−24.111	−19.633	−4.182	−22.987	−19.064
Guaiol	−4.641	−35.99	−27.375	−4.347	−37.28	−29.813
Isoaromadendrene Epoxide	−4.756	−25.101	−18.951	−3.816	−22.676	−18.632
Isocaryophyllene	−3.539	−26.62	−21.58	−3.661	−21.177	−17.684
p-Menth-1-en-4-ol	−4.527	−27.951	−21.226	−4.932	−28.877	−21.672
Selina-3,7(11)-diene	−3.842	−23.712	−19.036	−3.646	−18.916	−15.326
Trans-Pinocarveol	−4.574	−31.661	−24.556	−5.264	−30.66	−23.307
Valencene	−4.471	−25.337	−19.725	−3.762	−21.886	−18.662
Juniper Camphor	−4.741	−29.849	−22.429	−3.89	−23.471	−18.394
Acarbose	−6.507	−88.865	−64.575	−6.703	−91.874	−64.740

**Table 7:** ADME Properties of Compounds Predicted by QikProp.

	Mol MW	SASA	Donor HB	Accpt HB	QPlog Po/w	QPlog S	QPlog HERG	QPP Caco	QPlog BB	QPlog Kp	% Human Oral Absorption	Rule of Five
Juniper Camphor	222.37	500.489	1	0.75	4.058	−4.894	−3.541	3947.502	0.138	−2.179	100	0
alpha-Bisabolol	222.37	537.116	1	0.75	4.589	−4.927	−4.151	5855.642	0.041	−1.315	100	0
Bulnesol	222.37	485.138	1	0.75	4.128	−4.445	−3.138	5509.55	0.223	−1.812	100	0
Cubenol	222.37	486.509	1	0.75	4.145	−4.471	−3.276	6205.212	0.265	−1.68	100	0
alpha-Copaen-11-ol	220.354	473.907	1	0.75	4.014	−4.236	−3.178	5532.726	0.227	−1.717	100	0
Epiglobulol	222.37	466.713	1	0.75	3.988	−4.265	−2.781	5331.639	0.281	−1.946	100	0
gamma-Eudesmol	222.37	477.329	1	0.75	4.045	−4.3	−2.992	5043.834	0.194	−1.89	100	0
Guaiol	222.37	490.756	1	0.75	4.118	−4.55	−3.214	4678.352	0.158	−1.96	100	0
Isoaromadendrene Epoxide	220.354	438.021	0	2	2.504	−4.344	−2.417	9906.038	0.155	−0.872	100	0
alpha-Farnesene	204.355	563.12	0	0	6.992	−7.618	−4.727	9906.038	1.166	−0.674	100	1
Selina-3,7(11)-diene	204.355	469.974	0	0	5.382	−6.352	−3.296	9906.038	1.061	−1.393	100	1
Valencene	204.355	469.829	0	0	5.397	−6.33	−3.437	9906.038	1.011	−1.209	100	1
alpha-Gurjunene	204.355	460.168	0	0	5.191	−6.243	−2.947	9906.038	1.111	−1.515	100	1
Isocaryophyllene	204.355	466.076	0	0	5.314	−6.374	−3.138	9906.038	1.058	−1.402	100	1
alpha-Bulnesene	204.355	480.03	0	0	5.547	−6.506	−3.393	9906.038	1.097	−1.325	100	1
gamma-Gurjunene	204.355	465.548	0	0	5.303	−6.369	−3.113	9906.038	1.063	−1.316	100	1
beta-Eudesmene	204.355	464.71	0	0	5.306	−6.347	−3.23	9906.038	1.011	−1.228	100	1
beta-Farnesene	204.355	555.487	0	0	6.879	−7.563	−4.704	9906.038	1.105	−0.497	100	1
Cis-alpha-Bisabolene	204.355	500.398	0	0	5.912	−6.806	−3.796	9906.038	1.083	−1.036	100	1
alpha-Bergamotene	204.355	495.738	0	0	5.822	−6.682	−3.717	9906.038	1.106	−1.093	100	1
Caryophyllene	204.355	455.06	0	0	5.126	−6.221	−2.955	9906.038	1.039	−1.406	100	1
2-Pinen-4-one	150.22	365.729	0	2	1.92	−2.101	−2.509	3129.956	0.17	−2.426	100	0
alpha-Terpineol	154.252	396.847	1	0.75	2.962	−2.67	−3.051	4922.674	0.189	−1.793	100	0
p-Menth-1-en-4-ol	154.252	394.022	1	0.75	2.978	−2.625	−2.969	5690.276	0.244	−1.676	100	0
Camphol	154.252	361.633	1	1.7	2.079	−2.053	−2.207	4407.858	0.234	−2.107	100	0
Cis-Verbenol	152.236	375.213	1	1.7	2.124	−2.326	−2.766	4054.522	0.189	−2.085	100	0
Trans-Pinocarveol	152.236	371.828	1	1.7	2.14	−2.266	−2.599	4048.342	0.195	−2.084	100	0
Camphor	152.236	358.836	0	2	1.945	−1.99	−2.201	3964.567	0.259	−2.292	100	0
alpha-Pinene epoxide	152.236	339.822	0	2	1.399	−2.13	−1.707	9906.038	−0.042	−1.7	100	0
2-Fenchanol	154.252	369.822	1	1.7	2.207	−2.154	−2.387	4881.23	0.262	−2.021	100	0
beta-Linalool	154.252	431.483	1	0.75	3.14	−2.959	−3.59	5247.516	0.015	−1.421	100	0
D-Limonene	136.236	386.476	0	0	3.986	−4.004	−3.273	9906.038	0.833	−1.189	100	0
1,8-Cineole	154.252	377.4	0	0.75	2.461	−3.028	−2.596	9906.038	0.605	−0.892	100	0
beta-Cymene	134.221	384.991	0	0	3.842	−4.13	−3.723	9906.038	0.703	−0.966	100	0

## Data Availability

The original contributions presented in this study are included in the article. Further inquiries can be directed to the corresponding authors.
